# Intelligent Control of the Synthesis of Carbon Dots Used to Create a High-Precision Luminescent Nanosensor of Heavy Metal Ions in Water Using Machine Learning

**DOI:** 10.3390/molecules31213875

**Published:** 2026-10-27

**Authors:** Kirill Laptinskiy, Artem Guskov, Gavriil Kupriyanov, Galina Chugreeva, Alexey Vervald, Sergey Dolenko, Tatiana Dolenko

**Affiliations:** 1 Department of Physics, Moscow State University, 119991 Moscow, Russia; artemguskov99@mail.ru (A.G.); gavriil101@yandex.ru (G.K.); chugreeva.gn17@physics.msu.ru (G.C.); alexey.vervald@physics.msu.ru (A.V.); dolenko@srd.sinp.msu.ru (S.D.); tdolenko@mail.ru (T.D.); 2 Skobeltsyn Institute of Nuclear Physics, Moscow State University, 119991 Moscow, Russia

**Keywords:** carbon dots, nanosensors, machine learning, intelligent control, synthesis-properties-application problem

## Abstract

This article presents the results of solving a “Synthesis-Properties-Application” problem: finding optimal conditions for the hydrothermal synthesis of carbon dots from citric acid and ethylenediamine to create a photoluminescent nanosensor for the heavy metal salts Co(NO_3_)_2_ and Cu(NO_3_)_2_ in water, providing the highest sensitivity during quantitative analysis. The problem was solved in two stages. In the first stage, machine learning methods were used to estimate the errors in solving the inverse problem of determining the concentrations of Cu^2+^ and Co^2+^ ions in water from carbon dot photoluminescence excitation–emission matrix. The average solution error (mean absolute error—MAE) was 0.55 mM for Co^2+^ and 0.62 mM for Cu^2+^. In the second stage, optimal conditions for the synthesis of carbon dots with photoluminescence most sensitive to changes in the concentrations of Co^2+^ and Cu^2+^ ions in water were determined by analyzing the constructed approximation models of the dependence of the ion concentration determination error on the carbon dot synthesis parameters. The following values were obtained: a precursor ratio of CA:EDA = 1:2, a temperature of 150–155 °C, and a reaction time of 210–230 min. It was shown that carbon dots, effective as nanosensors for determining the concentration of Co^2+^ and Cu^2+^ cations in water, are formed mainly in the third stage of hydrothermal synthesis. The conclusions regarding the obtained optimal synthesis conditions were experimentally validated.

## 1. Introduction

Optical nanosensors based on nanoparticles whose luminescence is sensitive to changes in environmental parameters are currently being actively developed. Carbon dots (CDs) are among the most promising nanoparticles with such properties. CDs are a class of nanosized carbon particles that exhibit intense, stable luminescence, dispersibility in water, the ability to be doped with various atoms, and the ability to modify their surface to alter their optical properties [1,2]. Due to these properties, CDs hold great promise for use as optical nanosensors, theranostic agents, visualization tools, and other agents in various fields of science and technology [1,3,4,5,6,7,8,9,10].

In many areas, CDs are widely used as photoluminescent nanosensors of various characteristics and environmental conditions [4,5,6,7,8,9,10]. In particular, the photoluminescence (PL) spectra of CDs can be used to identify and determine the concentration of dissolved ions, including heavy metal cations [8,9,10], as well as organic analytes—for example, tyrosine-derived CDs have been used as a highly sensitive sensor for nitroxynil in food, urine, and live cells [11]. The complexity of developing CD nanosensors lies in the fact that, on the one hand, environmental parameters such as temperature, acidity, dissolved ions, and biomacromolecules alter CD luminescence in different ways [12,13,14,15], while on the other hand, the luminescence itself depends on the conditions of nanoparticle synthesis [1,2,9,16]. Therefore, nanosensorics is currently faced with a complex and new task of the “Synthesis-Properties-Application” type: the creation of a multimodal luminescent CD nanosensor for the high-precision determination of concentrations of specific environmental parameters/heavy metal ions in specific applications.

Monitoring ion composition, wastewater treatment, and monitoring the composition of food and beverages are both global and enterprise-specific problems for each industrial, agricultural, and food enterprises [17,18]. Each industry—pulp and paper, chemical, metallurgy, natural reservoirs, and beverages—has its own set of specific metal ions used in production, the concentrations of which should be simultaneously measured in real time when organizing closed-loop water recycling systems, discharging water into rivers, conducting environmental monitoring of reservoirs, or performing beverage quality control. Therefore, it is advisable to develop a CD-based sensor that provides high-precision measurement of specific ion concentrations for a specific situation—specific metal ions with known concentration ranges for each of them in the recycled water of a specific enterprise, maximum permissible concentrations (MPCs) for discharged or natural waters, MPCs for beverages, etc.

In recent years, CD-based fluorescent sensors for heavy metal ions have developed rapidly, with efforts directed at increasing their sensitivity, selectivity, and reliability. The most common approach is “turn-off” detection, in which analyte cations quench the PL of CDs through static or dynamic quenching or the inner filter effect [12,14,15]; highly selective CD-based probes of this type have been reported, in particular, for Cu^2+^ and Co^2+^ in water [19]. To reduce the influence of instrumental drift and probe concentration, ratiometric probes are being developed, in which the ratio of an analyte-responsive emission band to a reference band serves as the analytical signal [20,21]. However, real water samples usually contain several metal ions at once, and single-channel CD-based probes, whose PL is quenched by many transition metal cations, suffer from cross-sensitivity. This has stimulated the development of multiplexed luminescence detection platforms, which are attracting growing interest in various fields; for instance, an electrochemiluminescent DNA nanosphere integrated into microfluidic arrays detected several bacterial pathogens within 45 min [22]. For metal ions, such platforms are mainly fluorescent sensor arrays composed of several types of CDs with different surface chemistries or emission colors [23], or single CD-based sensors whose multichannel response is processed by machine learning methods to discriminate the ions and determine their concentrations in mixtures [24]. Our previous studies [8,10] follow the same multiplexed concept: the PL spectra of one type of CDs, processed by neural networks, serve as a multichannel signal for simultaneously determining several ion concentrations without fabricating an array of different nanoparticles. At the same time, these approaches, as a rule, optimize the sensor at the stage of signal acquisition and processing, while the CDs themselves are synthesized under empirically chosen conditions; tailoring the synthesis conditions to maximize the accuracy of multiplexed quantitative analysis remains practically unexplored.

The emphasis in solving “Synthesis-Properties-Application” problems shifts to determining the optimal synthesis parameters for obtaining nanoparticles with predetermined properties, for example, CDs with PL that is most sensitive to changes in the concentrations of specific ions. As follows from the literature [1,2,9,16], by widely varying precursors and synthesis conditions, it is possible to obtain CDs with a variety of characteristics and physicochemical properties, including luminescence. Obviously, for the hydrothermal method, the presence of complex dependencies of the properties of the resulting nanoparticles on a variety of different synthesis conditions, on the one hand, is one of the advantages, determining its flexibility and allowing for wide variation of the properties of the resulting CDs. However, on the other hand, the large number of these dependencies significantly complicates the search for optimal conditions for the targeted synthesis of nanoparticles. Due to such complexity and the multiparametric nature of the problem, to solve a problem of the “Synthesis-Properties-Application” type, it is advisable to use machine learning methods (MLM), which have enormous potential in the tasks of identifying hidden correlations in multiparametric systems and which have already proven themselves to be a promising and universal approach to solving problems addressed by the authors of this article [8,10,25].

In recent years, interest in searching for optimal conditions for the synthesis of carbon dots using MLMs to obtain nanoparticles with desired characteristics has increased sharply [26,27,28,29,30,31,32,33,34,35,36,37,38,39,40]. Primarily, the problem of obtaining CDs with intense PL or a high luminescence quantum yield (LQY), i.e., problems of the “Synthesis-Properties” type [26,27,28,29,30] was addressed. The authors of [29] used the XGBoost method to determine the PL quantum yield of CDs, with the input features being the volumes of ethylenediamine (EDA) and p-dihydroxybenzene precursors, the heating rate, the temperature, and the reaction time. It was found that the value of the PL quantum yield of the obtained nanoparticles depends most strongly on the EDA volume. Moreover, this dependence is nonlinear: excessive addition of EDA leads from stimulation of the synthesis reaction to passivation of the CD surface and a slowdown of the reaction. Based on the results of algorithm training, the synthesis conditions that produced CDs with an LQY of 39.3% were determined. In the paper [30], the XGBoost method was also used to predict the maximum intensity and the position of the PL maximum of CDs synthesized from p-benzoquinone (PBQ) and EDA and dispersed in different solvents. The input features for training the algorithm were the mass of PBQ, the volume of EDA, the reaction time, and two independent components obtained by principal component analysis based on the solvent characteristics (dielectric constant, viscosity, density, melting point, boiling point, and refractive index). Taking into account the influence of feature importance on the predictions of the model [31], the authors of [30] found that the optical properties of the synthesized CDs are most influenced by the volume of EDA and the mass of PBQ, and showed that using the trained XGBoost algorithm, it is possible to determine the maximum intensity and the position of the PL maximum with a coefficient of determination greater than 0.96.

The authors of [32,33,34,35] used various data processing methods and classification models to systematically analyze the effects of key synthesis conditions, including temperature, reaction time, and starting material mass, on the optical properties of CDs and optimize these synthesis conditions. By applying the XGBoost model with the classification capabilities of PCA and logistic regression to 151 experimental datasets, the synthesis conditions for obtaining CDs with red fluorescence characteristics were determined in [32]. Polynomial regression and random forest (RF) also showed high potential in optimizing the synthesis conditions of CDs to obtain the desired nanoparticle characteristics [29,33,34,35]. Chen et al. achieved 80% accuracy with the random forest model applied to create a multivariate system for determining the optimal values of the PL maximum wavelength, LQY, and Stokes shift (Δλ) of CDs in different solvents [35].

The authors of [30] used support vector machines (SVM), k-nearest neighbors (KNN), random forests, decision trees (DT), extreme gradient boosting (XGBoost), and one-dimensional convolutional neural networks (1D-CNN) to determine and optimize the optical properties of CDs. Based on 170 sets of synthesis conditions (precursor type, reaction time, and reaction temperature) borrowed from different papers, the authors used the aforementioned MLMs to identify complex nonlinear relationships between the synthesis conditions and the optical properties of CDs. Experimental verification confirmed that the set of synthesis conditions obtained using 1D-CNN provided the optimal optical characteristics of CDs for *HeLa* cell imaging. Tuchin et al. established a correlation between the red and near-infrared (NIR) emission of CDs and the synthesis conditions using multivariate linear regression and the KNN model [37]. Using a database of 127 samples, the influence of various precursors and solvents on the formation of optical centers in CDs was investigated. These results open new perspectives for the optimization of red-emitting CDs for biomedical applications. Senanayake et al. combined an artificial neural network with a hybrid classification–regression model and applied this construct to 407 literature datasets—synthesis condition databases—to determine the spectral characteristics of CD fluorescence [38]. Their results showed that the types of precursors and solvents have a much greater impact on the fluorescence characteristics than the reaction temperature and duration.

There are a number of publications presenting the results of successfully solving the “Synthesis-Properties-Application” problem [39,40]. The authors of [39] used SVM, KNN, RF, and XGBoost to predict the antimicrobial activity of CDs doped with L-lysine (ε-poly(L-lysine)). MLM algorithms were trained to solve the binary classification problem based on 17 input features, the most important of which were the size of nanoparticles, the mass of precursors, the number of carbon atoms in the precursors, and the zeta potential of CDs. The XGBoost algorithm provided the best solution to the classification problem, with an accuracy of 78.3%. CDs, due to their exceptional anticorrosion properties and environmental compatibility, are considered promising environmentally friendly corrosion inhibitors of the new generation. The authors of [40] analyzed 102 sets of CD synthesis conditions and the corresponding data on their anticorrosive properties using RF and identified nonlinear relationships between the hydrothermal synthesis parameters and their anticorrosive properties. Based on the obtained relationships, the problem of optimizing the synthesis conditions was solved using a genetic algorithm. The CDs synthesized under the identified conditions demonstrated an anticorrosive efficiency of up to 92.3%, with an experimental error of less than 3% compared to the results obtained using the RF model predictions, confirming the accuracy and reliability of the proposed approach [40].

However, successful solutions to the “Synthesis-Properties-Application” problems using machine learning methods require the availability of representative training samples, the preparation of which is often associated with significant time and financial costs, especially when solving inverse problems (IPs) of optical spectroscopy (OS). Consequently, researchers may be limited in the number of investigated CD synthesis conditions, which, in turn, complicates the production of nanoparticles with the desired properties. One approach that allows for a reduction in the requirements for the volume of available experimental data is the use of transfer learning methods [41], based on the use of knowledge obtained from solving more general problems with large volumes of data (initial problems) to solve more specialized problems with limited samples (target problems). This approach is based on the assumption that the data from the initial problem contains general patterns that retain their informative value for the target problem.

Transfer learning methods are increasingly used to solve various applied problems, including spectroscopic ones, to optimize the processes of analyzing experimental data [8,42,43,44,45,46,47]. For example, in [42], the Shotgun Transfer Learning approach was proposed, which made it possible to increase the efficiency of predicting the properties of materials (organic molecules, polymers, and inorganic compounds) under conditions of a limited volume of labeled data by transferring knowledge between models pre-trained on different sets of spectral data. The authors of [43] first simulated the Raman spectra of protein structures using semi-empirical methods of quantum chemistry, which allowed for the efficient training of deep learning models on large synthetic datasets. These pre-trained models were then further trained on a smaller experimental Raman spectroscopy dataset, including the spectra of bacteria. A comparative analysis with deep learning models and the PCA-LDA method showed that transfer learning significantly reduces computational costs while maintaining performance comparable to that of models trained from scratch in the context of real-world biosensors. The authors of [44] demonstrated the high efficiency of transfer learning by first training convolutional neural networks on openly published databases of Raman spectra of pathogenic bacteria and then further training them on Raman spectra of real healthy and cancerous tissue samples. Articles [45,46] demonstrated the successful application of transfer learning in NIR spectroscopy used to monitor the degree of chemical spoilage of meat [45] and in the use of pill spectra to predict the content of active pharmaceutical ingredients [46]. In the research of the authors of this paper, an important role was played by the methods of transfer learning used in solving various OS IPs [47], including in the development of a photoluminescent multimodal nanosensor based on carbon dots for the transition from solving a six-parameter to a more complex seven-parameter inverse problem of optical spectroscopy [8].

In solving spectroscopic problems, one of the challenges is identifying informationally useful regions of spectra, which are usually recorded with high spectral resolution. As a preprocessing method for two-dimensional data and images, the use of moments is a common approach to compressing the dimensionality of input data while preserving key characteristics of shape, intensity, and spatial distribution. This approach has a long history of development. Teh and Chin [44] systematically analyzed the effectiveness of different types of moments for image compression and noise filtering. In optical spectroscopy, a similar approach has been successfully applied to compress photoluminescence excitation–emission matrices (EEMs). Wang et al., using Zernike moments and stepwise linear regression with a feature selection algorithm, obtained a solution superior in quality to reference approaches—the application of partial least squares (PLS) and its modification to pure PL EEMs [48]. Similarly, Chen et al. used Zernike moments to efficiently extract features from the PL EEM of algae, which, in combination with gradient boosting methods, allowed for rapid and accurate determination of microbial concentrations [49]. In this study, the decomposition of PL EEMs into a classical centered power polynomial basis is used.

This study presents the results of a solution to the problem of determining the conditions for the hydrothermal synthesis of luminescent carbon dots that will ensure the lowest error in determining ion concentrations in water using photoluminescence spectra. Using MLMs, this article addresses the problem of determining the precursor ratio, temperature, and reaction time for the hydrothermal synthesis of CDs to produce a luminescent nanosensor capable of simultaneously measuring the concentrations of Cu^2+^ and Co^2+^ ions in water with high accuracy.

## 2. Results

### 2.1. Samples Characterization

All CD samples (diluted to a concentration 5 mg/L) were clear, colorless solutions with a pH of 6.2–6.9. The hydrodynamic sizes of all studied samples were virtually identical: the maximum in the size distribution was located around 0.67 nm, with its tail reaching 1.5–3 nm for different samples (Figure 1a). This maximum of the size distribution corresponds to the molecular luminophore (presumably IPCA [50]), while the tail indicates that these luminophores are linked into a slightly larger structure by polymers of EDA and CA, which is consistent with the polymer structure of the CDs used. The values of the zeta potentials of the samples depended on the ratio of the precursor concentrations: for CDs from CA:EDA = 1:1, the distribution maximum was located in the region of −1.5 ± 1.6 mV; for CA:EDA = 1:2—−4.1 ± 1.4 mV; and for higher ratios—−17 ± 3 mV. The half-width at half-height of all distributions was about 6 mV (Figure 1b).

FTIR absorption spectroscopy of the CD samples (Figure 1c) showed that all CDs contained a virtually identical set of bonds and surface groups: (C=O)-N, C=C, CH_x_, OH, N=O, and NO_2_. The IR spectra of the CD samples from CA:EDA 1:1 showed vibrations of the C-O and C=O bonds of the carboxyl groups (-COOH).

### 2.2. Photoluminescence Spectroscopy of Aqueous Solutions of Carbon Dots

PL EEMs were obtained for aqueous solutions of each of the 74 synthesized CD types with a concentration of 5 mg/L in the absence of salts and for all prepared aqueous CD solutions with Co(NO_3_)_2_ and Cu(NO_3_)_2_ salts.

Figure 2 shows the PL spectra of aqueous solutions of all 74 CD types synthesized under various conditions, obtained with excitation at 350 nm. The observed differences in the spectra indicate a significant dependence of the CD PL on their synthesis conditions— the ratio of precursors, temperature, and reaction time of the hydrothermal synthesis. The ratio of precursors has the greatest impact (Figure 2).

Figure 3 shows, as an example, the PL excitation–emission matrices of aqueous CD solutions with a concentration of 5 mg/L in the absence of salts (Figure 3a) and in the presence of Co(NO_3_)_2_ and Cu(NO_3_)_2_, each with a concentration of 4 mM (Figure 3b). The selected matrices correspond to CDs synthesized at the citric acid: ethylenediamine (CA:EDA) = 1:2 precursor ratio, a temperature of 200 °C, and a reaction time of 180 min. The presented PL maps of CDs are typical PL maps for all CDs synthesized by the hydrothermal method from CA and EDA and indicate significant quenching of CD fluorescence by metal ions in the solution. The quenching of CD fluorescence by metal ions in the solution is characteristic of all CDs, though the particular EEM intensities and shapes differ (Figure 2). According to our previous study [14] regarding the mechanisms of the PL quenching of a similar sample (EDA:CA 1:1, 190 °C, 120 min) by different metal ions Me^n+^, this quenching is more than 95% dynamic in nature; the remaining quenching is attributed, in the case of Fe3^+^, to the inner filter effect (IFE), and in the case of Cr^3+^, to a combination of the IFE and a type of static quenching associated with the deprotonation of surface groups resulting from changes in the pH of the samples.

All obtained results from photoluminescence spectroscopy of aqueous CD solutions in the absence and in the presence of salts demonstrate the need to find optimal synthesis conditions for obtaining CDs with the desired optical properties; in our case, CDs with PL that is most sensitive to changes in the concentrations of Cu^2+^ and Co^2+^ cations in water.

### 2.3. Application of Machine Learning Methods at Stage 1 of Solving the “Synthesis-Properties-Application” Problem. Error Assessment

#### 2.3.1. Application of Convolutional Neural Networks

Seventy-four CNNs were trained on the spectral dataset, one for each of the 74 CD types. Table S2 presents the mean absolute error (MAE) values for simultaneous ion concentration determination obtained by each of these 74 CNNs on the test dataset.

Each CNN had two convolutional layers, an initial learning rate of 0.001, and a regularization coefficient of 0.01. From this set of 74 trained CNNs, a neural network was selected that provided the minimum MAE values for determining the concentrations of the studied Co^2+^ and Cu^2+^ cations. Table 1 lists the minimum MAE values for determining the concentrations of Co^2+^ and Cu^2+^ obtained with this network and the synthesis conditions for the type of CDs on whose spectral data this CNN was trained.

#### 2.3.2. Application of Multilayer Perceptron and Its Transfer Learning

The results of solving the inverse problem of autonomous determination of heavy metal ion concentrations in aqueous solutions using the excitation–emission matrices of the PL of CDs and the multilayer perceptron (MLP) on the full dataset (L-MLP) are presented in Table S3.

Similar tables were generated for all other problem formulation options: S-MLP, TL_NB_R, TL_NB_RTT, and TL_S-MLP. (Please refer to the Sections Multilayer Perceptron and Transfer Learning of Multilayer Perceptron for an explanation of these options.) These data will be used later to train the approximation model.

Based on the obtained data, error range diagrams for determining the concentrations of Co^2+^ and Cu^2+^ ions were constructed for all inverse problem formulations (Figure 4). Each diagram reflects the statistical distribution of the MAE across all CD types.

Table 2 presents the minimum, median, and average errors in determining the concentrations of the ions under study, calculated for all types of CDs, for each variant of the problem statement.

As can be seen from Figure 4 and Table 2, the minimal error in ion concentration determination is achieved by using the multi-stage transfer learning approach (TL_S-MLP). The minimal median and average error values calculated for all types of CDs were also obtained using TL_S-MLP. An exception is the average error in Cu^2+^ concentration determination: when training only on data from one type of CDs (S-MLP), significantly lower maximum error values were observed compared to those obtained using the transfer learning approach (TL_S-MLP), which resulted in a lower average error value for the S-MLP problem.

It should be noted that during the step-by-step additional training of the model, a consistent decrease in the minimum, median, and average error values calculated for all CD types is observed (Table 2). When training on the full dataset (L-MLP), the highest minimum, median, and average error values for determining salt concentrations are observed. This is due to inconsistencies in the data: identical output parameter values—ion concentrations—correspond to different PL spectra of different CD types. When training only on data from a single CD type (S-MLP), the MLP provides an acceptable solution to the problem but is inferior in accuracy to the transfer learning approach (TL_S-MLP).

Table 1 above presents the minimum MAE values for determining the concentrations of the ions under study, obtained using the TL_S-MLP multi-step transfer learning approach, as well as the corresponding CD synthesis conditions for which these values were obtained. It should be noted that the CD synthesis conditions for which the minimum MAE values are obtained are within the range of conditions that ensure a high quantum yield of photoluminescence for the CDs [16]. Since a high LQY is key to using CDs as ion nanosensors, such synthesis conditions can be considered potentially optimal.

Thus, the obtained results demonstrate that the TL_S-MLP multi-step transfer learning approach is capable of providing an effective solution to the posed inverse problem: for certain types of CDs, the relative error does not exceed 10%.

#### 2.3.3. Application of the Method of Moments

The minimum MAE values for determining the concentrations of the studied Co^2+^ and Cu^2+^ ions, obtained using linear regression and gradient boosting (LR/GB) (see the Section Application of the Method of Moments), applied to the CD PL EEMs compressed using the method of moments (Section Application of the Method of Moments), are given above in Table 1.

### 2.4. Stage 2. Construction of an Approximation Model of the Dependence of the MAE for Determining the Ion Concentration on the Synthesis Conditions of the CDs

The initial data for training the approximation model were the MAE values obtained by the MLP using a multi-stage transfer learning procedure (TL_S-MLP). This approach proved to be the most effective one in solving the inverse problem of determining the concentrations of the studied ions (see below, Section 3.1).

The functional surfaces were constructed by applying the approximation model to a denser grid of synthesis conditions than the experimental one: temperatures from 80 to 220 °C with 2 °C increments, reaction times from 0 to 420 min with 5 min increments, and CA:EDA ratios corresponding to the experimental values with the addition of a 1:15 ratio. The ranges of synthesis conditions were slightly extended beyond the experimental grid to evaluate the extrapolation capabilities of the approximator.

To visualize the functional surfaces, one of the four variables—precursor ratio, temperature, reaction time, or MAE—had to be fixed. In the presented surfaces, the precursor ratio was fixed (indicated in the caption for each graph), and the MAE value was displayed using a color scale. Additionally, the graphs contain circles corresponding to the experimental values of the CD synthesis conditions (74 points). The color of the circles reflects the MAE value obtained using the MLP for the corresponding CD type during the error estimation stage. The error values indicated in the circles were used as training data for the approximation model. The black solid and white dotted lines delimit the regions of high values of the LQY and of the integrated PL intensity of the CDs, respectively.

The results of the neural network approximation of the MAE for determining the concentration of Co^2+^ ions in aqueous solutions depending on the conditions of hydrothermal synthesis of CDs are presented in Figure 5; those for the concentration of Cu^2+^ ions are presented in Figure 6.

## 3. Discussion

### 3.1. Comparison of the Quality of the Solution of the Inverse Problem of Estimating the Error in Determining the Concentration of Co^2+^ and Cu^2+^ Ions from the PL EEM of Carbon Dots Using Different Machine Learning Methods (Stage 1)

Table 1 above presents the minimum error estimates for the concentrations of the ions under study, obtained using CNN, MLP using a multi-stage transfer learning approach (TL_S-MLP), and LR/GB applied to CD PL compressed using the method of moments (MM), as well as the corresponding CD synthesis conditions for which the presented values were obtained.

As follows from the presented results, the smallest minimum error values for determining the concentrations of heavy metal ions in aqueous solutions using the EEM of CDs, calculated for all CDs, were obtained using LR/GB on PL matrices compressed using MM.

A stability analysis was performed for all error estimates obtained using CNN, TL_S-MLP, and LR/GB (MM).

Table 3 presents the mean, median, and minimum errors in determining the concentrations of the studied ions, calculated using the PL EEM for all types of CDs using CNNs, TL_S-MLP, and LR/GB (MM). The minimum error in determining ion concentrations was achieved using LR/GB (MM). However, the minimum median and mean error values for the Co^2+^ ion were achieved using the multi-stage transfer learning approach (TL_S-MLP). For the Cu^2+^ ion, the median error was lower using LR/GB (MM). Despite the relatively low minimum error in solving the problem achieved by CNNs, they are characterized by higher median and mean error values, which are significantly inferior to the corresponding values obtained using other methods.

Figure 7 shows the error range diagrams for determining the concentration of Co^2+^ and Cu^2+^ ions using all the inverse-problem-solving methods used (CNN, TL_S-MLP, and LR/GB (MM)). Each diagram reflects the statistical distribution of errors for all types of CDs.

Based on the obtained results, it can be concluded that the most effective method for determining Co^2+^ and Cu^2+^ ion concentrations from the PL EEM of carbon dots is the multi-stage transfer learning approach (TL_M-MLP). This method provides the most stable solution quality for the inverse problem, as evidenced by the low median and mean error values calculated from the spectral data of all types of CDs, and significantly lower values corresponding to the third quartile and 95th percentile compared to other methods (Figure 7).

### 3.2. Stage 2. Determination of Optimal Conditions for the Synthesis of CDs with PL Most Sensitive to Changes in the Concentrations of Co^2+^ and Cu^2+^ Ions in Water

#### 3.2.1. Evaluation of the Areas of Optimal Conditions for the Synthesis of the Most Sensitive CD Nanosensors Based on the Results of Applying Three Methods: CNN, TL_S-MLP, and LR/GB (MM)

Table S4 presents the five lowest MAE values for determining the concentrations of the ions under study, obtained using all three methods: CNN, TL_S-MLP, and LR/GB (MM), as well as the corresponding CD synthesis conditions for which the given values were obtained. Figure 8 shows the locations of these CDs, which provide minimal errors in determining the concentrations of Co^2+^ and Cu^2+^, respectively, in the three-dimensional space of nanoparticle synthesis conditions. The figure shows the five CDs with the lowest MAE for each of the three methods for solving the problem—CNN, TL_S-MLP, and LR/GB (MM). For each point, its projection onto the time–temperature plane is also shown.

The following conclusions can be drawn from the data obtained in Table S4 and Figure 8:

- Most of the CDs for which the best inverse problem solution quality was achieved as a result of applying all three methods were synthesized with CA:EDA precursor ratios of 1:2 and 1:4.
- Most of the CDs providing the best MAE for Co^2+^ determination were synthesized at temperatures above 140 °C and were distributed across the entire studied range of CD synthesis times, regardless of the solution method.
- CDs providing the best MAE for Cu^2+^ determination, in contrast, are localized either in the region of high temperatures and short reaction times or in the region of relatively low temperatures and long synthesis times.

A more accurate determination of the optimal synthesis conditions for CDs with PL most sensitive to changes in Co^2+^ and Cu^2+^ ion concentrations in water was obtained by analyzing the constructed approximation models of the dependence of the MAE for determining ion concentrations on the CD synthesis parameters.

#### 3.2.2. The Main Stages of Hydrothermal Synthesis of Carbon Dots from Citric Acid and Ethylenediamine

Simultaneously with the determination of the optimal conditions for the synthesis of CDs with PL most sensitive to changes in the concentrations of Co^2+^ and Cu^2+^ ions in water, the results of the analysis of the constructed approximation models of the dependence of the MAE for determining ion concentrations on the CD synthesis conditions (Figure 5 and Figure 6) were compared with the results of the authors’ study of the stages of CD synthesis by the hydrothermal method from citric acid and ethylenediamine [16].

In [16], a database of 392 CD samples was obtained, synthesized by the hydrothermal method from ethylenediamine and citric acid precursors with parameters varying within the following ranges: CA:EDA ratio—from 1:0 to 1:20, temperature—from 80 to 200 °C and reaction time from 30 to 360 min. Optical density spectra, fluorescence EEM, and FTIR absorption spectra were obtained for all samples, and the LQY and luminophore intensity (Ilum) values of CDs were calculated at an excitation wavelength of 350 nm. A combined analysis of optical and FTIR spectroscopy results allowed us to identify the key stages in the hydrothermal synthesis of CDs from EDA and CA in terms of the structural transformation of the reaction products and the formation of luminophores.

(1) Initial stage of synthesis. Precursors are mixed at room temperature, EDA reacts with CA, and polymerization of the precursors begins. There is no fluorescence of the reaction products.
(2) Initial stage of luminophore formation. CDs synthesized from any CA:EDA ratio at temperatures up to 100 °C for up to 60 min exhibit very weak fluorescence and low LQY (units of %).

As a result of the hydrothermal reaction of CA and EDA precursors (if both are present) at a temperature of 120–130 °C and a reaction time of 120–150 min (depending on the precursor concentration ratio), the primary L350 luminophores begin to form, with an excitation maximum at 350 nm. The LQY of this luminophore at an excitation wavelength of 350 nm is about 98%.

It should be noted that at CA:EDA ratios of 1:2 and 1:4, at the studied temperatures and reaction times, significantly more of the primary luminophore was formed than at other CA:EDA ratios.

(3) Sample carbonization stage. With a consistent increase in temperature to 160–170 °C and synthesis time to 240–290 min, the active formation of L350 luminophores continues, but the carbonization of the CDs begins, resulting in a decrease in the LQY of the CDs, despite the continued increase in the luminophore intensity of the samples.
(4) Luminophore decomposition stage. At temperatures and synthesis times greater than 160–170 °C and 240–290 min, respectively, the rate of further carbonization of the samples outpaces the rate of synthesis of new L350 luminophores, leading to graphitization of the CDs and a decrease in the number of L350 luminophores. This leads to both a decrease in the luminescence intensity and a decrease in the LQY of the CDs.

It should be noted that the transition between the synthesis stages is smooth, with each of the four stages occupying a wide region in the temperature and time coordinates of synthesis. Stages 2 and 3 produce the majority of the L350 luminophores, which provide the stable, intense PL of the CD samples and high LQY. Clearly, these CDs are the most effective nanosensors for detecting ions in water. However, stages 2 and 3 occupy a large region in the three-dimensional synthesis parameter space considered here. Therefore, to develop CD-based nanosensors with high selectivity and sensitivity of their PL to specific ions, a more precise determination of the synthesis conditions for such CD nanosensors is necessary. For this purpose, the ranges of optimal CD synthesis conditions were determined using an approximation model of the dependence of the MAE for determining ion concentrations on the CD synthesis conditions.

#### 3.2.3. Analysis of the Approximation Model of the Dependence of the MAE for Determining the Ion Concentration on the Synthesis Conditions of the CDs

As follows from the analysis of the constructed approximation models of the dependence of the MAE for determining ion concentrations on the CD synthesis conditions (Figure 5 and Figure 6), the situation is qualitatively the same for both metal ions. For the studied precursor ratios, all surfaces have two qualitative regions: a region of high MAE (red-brown) and a region of acceptable MAE (violet to yellow).

The regions of high MAE correspond to low temperatures or short synthesis times. According to the data presented in Section 3.2.2, at these reaction temperatures and times, an insufficient amount of primary luminophores is formed, which results in the low fluorescence intensity of CDs and, consequently, complicates the determination of the contribution of heavy metals to the fluorescence spectra.

Of greatest interest for nanosensors are the low-MAE regions (violet-blue regions in Figure 5 and Figure 6), which are most pronounced for CA:EDA precursor ratios < 1:10 (Figure 5 and Figure 6). At CA:EDA = 1:1, the experimentally synthesized CDs (round markers) are not present in the low-MAE region, so the MAE surface extrapolation result is unreliable. For CA:EDA = 1:2 and CA:EDA = 1:4, the experimentally synthesized CDs are present both in the low-MAE region and at the boundary between high and low MAE. Therefore, the MAE surface interpolation result for CA:EDA = 1:2 and CA:EDA = 1:4 can be considered reliable.

A combined analysis of the results of the study on the stages of CD hydrothermal synthesis and their effectiveness as nanosensors was conducted. The analysis consisted of comparing the ranges of temperature and time changes of nanoparticle synthesis in which CDs with the highest LQY and intense PL are formed (black and white polygons, respectively) and for which a region of low MAE is observed for CA:EDA = 1:2 and CA:EDA = 1:4.

The following conclusions were drawn for both Co^2+^ and Cu^2+^ ions studied:

- The temperature and reaction time values for the lower left boundary between the regions of high and low MAE are close to those at the beginning of the second stage of CD synthesis, when CDs with high LQY begin to form (lower left boundary of the black polygon).
- The boundaries of the regions of minimal MAE coincide with the boundaries of the regions of intense integrated PL (region with a white dotted boundary), corresponding to the parameters of the third stage of CD synthesis. Table 4 lists the CD synthesis conditions under which, according to the PL EEM, the concentrations of Co^2+^ and Cu^2+^ ions in water are determined with the lowest MAE.

Thus, carbon dots effective as nanosensors for determining the concentration of Co^2+^ and Cu^2+^ cations in water are formed primarily at stage 3 of hydrothermal synthesis.

Based on the obtained results (Table 1 and Table 4, Figure 5 and Figure 6), the optimal conditions for synthesizing CD nanosensors with photoluminescence most sensitive to both Co^2+^ and Cu^2+^ ions were determined: a precursor ratio of CA:EDA = 1:2, a temperature of 150–155 °C, and a reaction time of 210–230 min. With longer synthesis times and higher temperatures, the main L350 luminophores are destroyed (during the transition from stage 3 to stage 4 of CD synthesis), which is accompanied by a deterioration in nanosensor accuracy.

## 4. Materials and Methods

### 4.1. Materials and Synthesis of Carbon Dots

The nanosensors used were synthesized by the hydrothermal method from citric acid (CA, Acros Organics, Geel, Belgium) and ethylenediamine (EDA, Acros Organics, Geel, Belgium) precursors. The metal nitrates Co(NO_3_)_2_ and Cu(NO_3_)_2_ were of reagent grade (PanReac AppliChem, Barselona, Spain, Darmstadt, Germany). Double-distilled deionized water (Millipore Milli-Q water purification system, Darmstadt, Germany) was used as the solvent.

A detailed description of the hydrothermal synthesis of CDs is presented in [16]. In this study, a set of 74 CD samples synthesized from citric acid and ethylenediamine was obtained under different combinations of synthesis conditions (see the flowchart in Figure 9): the precursor ratio (CA:EDA) varied from 1:1 to 1:20, the temperature ranged from 100 °C to 200 °C in 20 °C increments, and the reaction time ranged from 30 to 360 min (see Table S2 for the parameters of the individual samples). These particular samples were chosen based on their QYs determined in [15] at the excitation wavelength of 350 nm being greater than 75%.

### 4.2. Preparation of Aqueous Solutions of Carbon Dots and Salts

Aqueous solutions of each type of CDs with a concentration of 5 mg/L and aqueous solutions of salts (Cu(NO_3_)_2_ and Co(NO_3_)_2_) with concentrations ranging from 0 to 6 mM in increments of 0.67 mM were prepared. Then, these solutions were mixed in such a way that the studied aqueous solutions contained CDs with a fixed concentration of 5 mg/L and Cu^2+^ and Co^2+^ cations with concentrations varying from 0 to 6 mM with a concentration step of 0.67 mM. The concentration of the NO_3_^−^ anion directly depended on the concentration of the cations and varied within the range of 0 to 24 mM. As a result, 100 samples were prepared for each type of CDs, amounting to a total of 7400 aqueous solutions of CDs with Co(NO_3_)_2_ and Cu(NO_3_)_2_ salts.

### 4.3. Experimental Methods

Hydrodynamic size and zeta-potential distributions were obtained for the representative samples using a Malvern ZetaSizer Nano ZS device (Malvern Panalytical, Malvern, United Kingdom). The pH values of the studied solutions were measured using an Aquilon I-500 pH meter (Aquilon, Moscow, Russia). IR absorption spectra of the samples were recorded using a Bruker INVENIO R FTIR spectrometer (Bruker, Ettlingen, Germany) equipped with an attenuated total reflectance attachment with a diamond crystal, with 15 μL of each sample placed on the crystal and dried under a constant flow of room air for 15 min.

Photoluminescence excitation–emission matrices were obtained for all 7400 samples using a Shimadzu RF-6000 spectrofluorimeter (Shimadzu Corporation, Kyoto, Japan). The samples were placed in quartz cuvettes with an optical path length of 1 cm. The signal was excited by radiation at 27 wavelengths, ranging from 280 to 410 nm in increments of 5 nm. PL spectra were recorded in the spectral range from 375 to 575 nm in increments of 1 nm (201 spectral channels) at a scan rate of 6000 nm/min with low sensitivity. Thus, the obtained PL EEM had dimensions of [27 × 201] = 5427 channels. Optical density spectra were obtained using a Shimadzu UV-1800 spectrophotometer (Shimadzu Corporation, Kyoto, Japan).

### 4.4. Using Machine Learning Methods to Solve the “Synthesis-Properties-Application” Problem

To solve the posed “Synthesis-Properties-Application” problem, machine learning methods were used. The primary objective of this study was to develop a model capable of approximating the dependence of ion concentration determination error on the CD synthesis conditions—precursor ratio, reaction temperature, and reaction time. The target variable values for training the approximating model were obtained by solving an inverse photoluminescence spectroscopy problem: determining the concentrations of the ions under study (including heavy metals) in aqueous solutions based on the measured PL spectra of each synthesized CD. As a result, the primary task was divided into two stages:

Stage 1. Error assessment: Solving the inverse problem of determining the concentrations of the studied salts in solutions based on the excitation–emission matrices of the CD fluorescence using MLMs separately for each type of CD.

Stage 2. Construction of an approximation model: Training a model describing the relationship between the error in determining the ion concentration and the CD synthesis conditions.

To solve the stated photoluminescence spectroscopy problem, the following MLMs were used: a convolutional neural network, a multilayer perceptron (MLP), and MLP transfer learning.

The mean absolute error (MAE) served as the metric for assessing the quality of the MLM application:

(1) $$MAE=\;\frac{1}{N}\sum_{i=1}^{N}\left|y_{i}-{\hat{y}}_{i}\right|$$

where *N* is the number of patterns, $y_{i}$ is the real value of the target variable, and ${\hat{y}}_{i}$ is the model response.

#### 4.4.1. Stage 1 of Solving the “Properties–Synthesis–Application” Problem. Error Assessment

All ML algorithms used the excitation–emission matrices of CD PL as input data. The input feature array had 5427 values (27 excitation wavelengths × 201 emission wavelengths); for processing by the MLP, it was stretched into a one-dimensional vector. Before use, each input array (the PL EEM of a single sample) was normalized to its maximum value (the maximum PL intensity). Preliminary studies have shown that this type of normalization provided minimal error in determining ion concentrations from normalized EEMs. The total database consisted of 7400 patterns.

The initial database was divided into training, validation, and test sets. Patterns from the training set were used to train the models, the validation set was used to prevent network overfitting, and the test set was used to evaluate model quality on independent data. For each of the 74 CD types, a separate test set comprising 16 patterns was formed—the PL EEMs of the 16 patterns of the same CD type. Thus, 74 independent test sets were formed. The spectral data for each CD type were divided into training and test sets not randomly, but according to a deterministic scheme, as shown in Figure 10. The concentrations of each of the two salts in the solutions varied from 0 to 6 mM in steps of 0.67 mM, which corresponded to an experimental concentration grid of 10 × 10 points. Circles indicate grid points included in the training dataset, and crosses indicate points assigned to the test set. The use of deterministic partitioning was necessary to ensure strict independence of the results from the selection of test sets, as well as to allow correct comparison of results between different IP statements.

A validation sample was randomly selected from the training dataset. The neural network performance was periodically assessed on this sample during training. Training was stopped after a specified number of epochs, after the error on the validation set had reached a minimum.

The neural networks were trained either on a set of 6216 patterns (74 × 100 − 74 × 16) or on subsets of 84 patterns (100 − 16) corresponding to the training data for a specific CD type. In the latter case, 74 neural networks were trained.

The output features for all applied machine learning algorithms were the cation concentrations of two salts: Co(NO_3_)_2_ and Cu(NO_3_)_2_. Simultaneous and autonomous determination of target parameters was used. During the autonomous determination, a separate model was built for each of the two parameters being determined.

As mentioned earlier, at stage 1 of the solution to the given problem, a convolutional neural network, a multilayer perceptron, and transfer learning of the MLP were used, as well as a special method of preprocessing multidimensional data based on the calculation of moments (hereinafter referred to as the method of moments, MM).

##### Convolutional Neural Networks

When selecting the CNN architecture for solving the stated problem, it was taken into account that the input patterns represented PL EEM intensities. In such matrices, the PL intensities in closely spaced spectral channels are significantly correlated. Therefore, two-dimensional CNNs were chosen (Figure 11), which have proven effective in processing data with ordered correlated features (e.g., images). A total of 74 CNNs were trained, with each CNN trained on subsets of 84 patterns corresponding to the training data for each of the 74 CD types. Since each model was trained on a small number of patterns, the model architecture was selected to minimize the number of trainable parameters: 1–2 convolutional layers with two channels and a filter size of 2; a max-pooling layer that reduces the PL matrix by a factor of 2 along each dimension; and a fully connected hidden layer containing 32 neurons. Simultaneous determination of both target parameters was used: the number of neurons in the output layer was equal to 2. The LeakyReLU activation function was used.

The learning rate started at 0.001 and was decreased by a factor of 5 if the MAE metric on the validation set did not decrease over 25 epochs (the minimum achievable learning rate was 10^−6^). Training was stopped if the error on the validation set did not decrease over 50 epochs.

During CNN training, the following parameters were used:

(1) The number of convolutional layers in the neural network architecture was 1 or 2.
(2) The regularization parameter during training varied from 10^−1^ to 10^−3^.
(3) The initial learning rate varied from 10^−3^ to 10^−6^.

##### Multilayer Perceptron and Transfer Learning of Multilayer Perceptron

To determine the concentrations of the studied heavy metal ions in aqueous solutions using the PL EEM of CDs, a neural network with a multilayer perceptron architecture was used. The model hyperparameters were as follows: the MLP architecture consisted of one hidden layer of 16 neurons; the transfer function for the hidden layer was sigmoidal; for the output layer, it was linear. Model training was performed using the Adam adaptive optimization algorithm.

Two variants of MLP training were used: (1) on a large training set of 6216 patterns for all CD types (L-MLP) and (2) on a small dataset—subsets of 84 patterns each, corresponding to the training data for each of the 74 CD types (S-MLP).

The network training termination criterion was exceeding a timeout of 500 epochs after the minimum mean squared error was reached on the validation set. The validation set was generated three times to reduce the influence of random factors on the results of the machine learning algorithms. The presented results were obtained by averaging the statistical indicators of three such models.

In Stage 1 of the solution to the stated problem, in addition to using the L-MLP and S-MLP, transfer learning (TL) of the neural network was used. It consisted of using a model trained to solve a data-intensive task (L-MLP) to solve a similar target task with less data (S-MLP) by adapting it to the new task through fine-tuning.

The process of adapting the L-MLP, trained on the full spectral dataset, to the problem for a specific (target) type of CDs was carried out in stages:

1. TL_NB_R: further training of the L-MLP model, pre-trained on the full dataset, on a sample including data from the target CDs, data from all CDs with the same precursor ratio as the target CDs, and data from all CDs closest to the target (neighboring) in terms of precursor ratio.
2. TL_NB_RTT: further training of the model pre-trained in the TL_NB_R stage on a dataset including data from the target CDs and data from neighboring CDs closest to the target in terms of the three synthesis conditions.
3. TL_S-MLP: further training of the model pre-trained in the TL_NB_RTT stage on data from the target CD only (S-MLP).

For different problem formulations (L-MLP, S-MLP, TL), including different stages of transfer learning (TL_NB_R, TL_NB_RTT, TL_S-MLP), the following model hyperparameters were varied: learning rate, L2 regularization coefficient, and validation set size. The values of the corresponding hyperparameters are presented in Table S1.

##### Application of the Method of Moments

Using moments as a preprocessing method for two-dimensional data and images is a common approach to reducing the dimensionality of input data while preserving key characteristics of the shape, intensity, and spatial distribution of images.

As noted earlier, the input feature vector for the PL EEM of carbon dots had 5427 values, while the training set for each CD type contained only 84 patterns (16 patterns were used for the test set). Therefore, we considered reducing the dimensionality of the input space. For this purpose, we used centered moments of the PL EEM, calculated using the formula:

(2) $$M^{a,b}=\iint \sqrt[{a+b}]{{\left(x-x_{0}\right)}^{a}\cdot {\left(y-y_{0}\right)}^{b}\cdot PL\left(x,y\right)}dxdy\;\approx \sum_{x,y}\sqrt[{a+b}]{{\left(x-x_{0}\right)}^{a}\cdot {\left(y-y_{0}\right)}^{b}\cdot PL(x,y)}$$

where $M^{a,b}$ are the moments of the PL EEM of the order (a, b);

a, b are the orders of the moments along the emission and excitation wavelength axes, respectively;

x, y are the values of the emission and excitation wavelengths, respectively;

$x_{0},\;y_{0}$ are the positions of the center of mass of the PL EEM along the emission and excitation axes, respectively.

Moments up to the 23rd total order (a + b ≤ 23) were used, allowing for the generation of 300 input features (this number of features was determined to be optimal during preliminary experiments). Linear regression and gradient boosting models were trained on the resulting feature space. Linear regression was chosen because it is the simplest model, with a minimal number of trainable parameters, and it is less prone to overfitting on a small sample. Gradient boosting was chosen as the most robust model, capable of describing nonlinear relationships. The choice between these models was made for each CD type using 10-fold cross-validation on the training set based on the minimum mean average error, after which the selected model was trained on the entire training set.

#### 4.4.2. Stage 2. Construction of the Approximation Model

To approximate the dependence of the error in determining the concentrations of the studied heavy metal ions on the CD synthesis conditions, an MLP was used due to its ability to reveal hidden relationships in multiparameter systems and to provide an accurate and smooth approximation of complex functional dependencies.

The input features for the MLP were the CD hydrothermal synthesis conditions: precursor ratio, temperature, and reaction time. The output feature (autonomous parameter determination) was the error (MAE) in determining the ion concentration (the target variable values for training the approximation model were obtained in Stage 1—the error estimation stage). Each training pattern corresponded to one CD type, so the training set size was 74 patterns.

The following hyperparameters of the approximation model were used: the MLP architecture had a single hidden layer consisting of 8 neurons; the transfer function for the hidden layer was sigmoidal, and for the output layer, it was linear; the learning rate was 0.1; and the momentum value was 0.5. Perceptron weights were optimized using stochastic gradient descent over 1000 epochs using the synthesis parameters and the MAE of all 74 CDs. The small number of neurons in the hidden layer ensured the necessary “rigidity” of the approximator and prevented model overfitting in the absence of early stopping on the validation set.

## 5. Approbation of the Method

To assess the correctness of the constructed approximation dependencies of the MAE in determining the concentrations of ions on the synthesis conditions of CDs, the obtained approximation models were tested. For this purpose, two types of CDs were synthesized 3 times (3 independent batches) under conditions determined by the approximators as optimal separately for each of the ions under study: CD1 at CA:EDA 1:2, temperature 154 °C, reaction time 215 min; CD2 at CA:EDA 1:2, temperature 152 °C, reaction time 220 min. For each batch and type of synthesized CD, 100 aqueous solutions were prepared with the addition of Co(NO_3_)_2_ and Cu(NO_3_)_2_ salts. The concentrations of ions in the prepared solutions varied within the same ranges as during the error assessment stage of the IP solution.

The testing was carried out using a multi-stage transfer learning approach. The training and test sets for the new target CDs were formed according to a similar scheme presented in Figure 10 (the test set included 16 patterns). The results of applying the multi-stage transfer learning approach, as well as the conditions for synthesizing the prepared CD samples, are presented in Table 5. The low MAE values obtained in the experiment turned out to be very close to the estimates of the approximation models.

## 6. Conclusions

This study examines the problem of finding optimal conditions for the hydrothermal synthesis of carbon dots from citric acid and ethylenediamine to create a luminescent nanosensor for the cations of heavy metal salts Co(NO_3_)_2_ and Cu(NO_3_)_2_ in water, ensuring the highest sensitivity during quantitative analysis. Ion concentrations in a solution were determined using machine learning methods based on the analysis of changes in the shape of a photoluminescence EEM of the carbon dots.

The problem was solved in two stages.

In the first stage, machine learning methods were used to estimate the errors in solving the inverse problem of determining the concentrations of Cu^2+^ and Co^2+^ ions in water from CD PL EEMs. It was shown that the most effective method for determining concentrations was the multi-stage transfer learning approach using a multilayer perceptron (TL_S-MLP), which provided the most stable solution quality for the inverse problem. The average solution error (MAE) was 0.55 mM for Co^2+^ and 0.62 mM for Cu^2+^.

In the second stage, optimal conditions for the synthesis of CDs with PL most sensitive to changes in the concentrations of Co^2+^ and Cu^2+^ ions in water were determined by analyzing the constructed approximation models of the dependence of the ion concentration determination error on the CD synthesis parameters. The following values were obtained: a precursor ratio of CA:EDA = 1:2, a temperature of 150–155 °C, and a reaction time of 210–230 min. It was shown that CDs, effective as nanosensors for determining the concentration of Co^2+^ and Cu^2+^ cations in water, were formed mainly in the third stage of hydrothermal synthesis.

An experimental validation of the conclusions regarding the optimal synthesis conditions obtained using an approximation approach based on machine learning was carried out. Two CD samples were synthesized with synthesis parameters corresponding to the minima of the approximation models for both ions. For each of them, 100 aqueous solutions with various combinations of the concentrations of the target ions were prepared, in a manner similar to that used to construct the approximation models. The inverse problem of determining the ion concentrations was solved based on the spectra of these solutions. The low error values obtained in solving the inverse problem turned out to be very close to the estimates of the approximation models for these synthesis parameters of CDs, which confirmed the effectiveness of the approach used to solve the problem of determining the optimal conditions for CD synthesis.

Thus, this study demonstrates the possibility and effectiveness of intelligent control over the synthesis of carbon dots for the creation of a luminescent nanosensor for heavy metal ions in water using machine learning. The results obtained from the development of “smart” synthesis of carbon dots contribute to further innovations in the field of multifunctional applications of carbon dots.

## Figures and Tables

**Figure 1:**
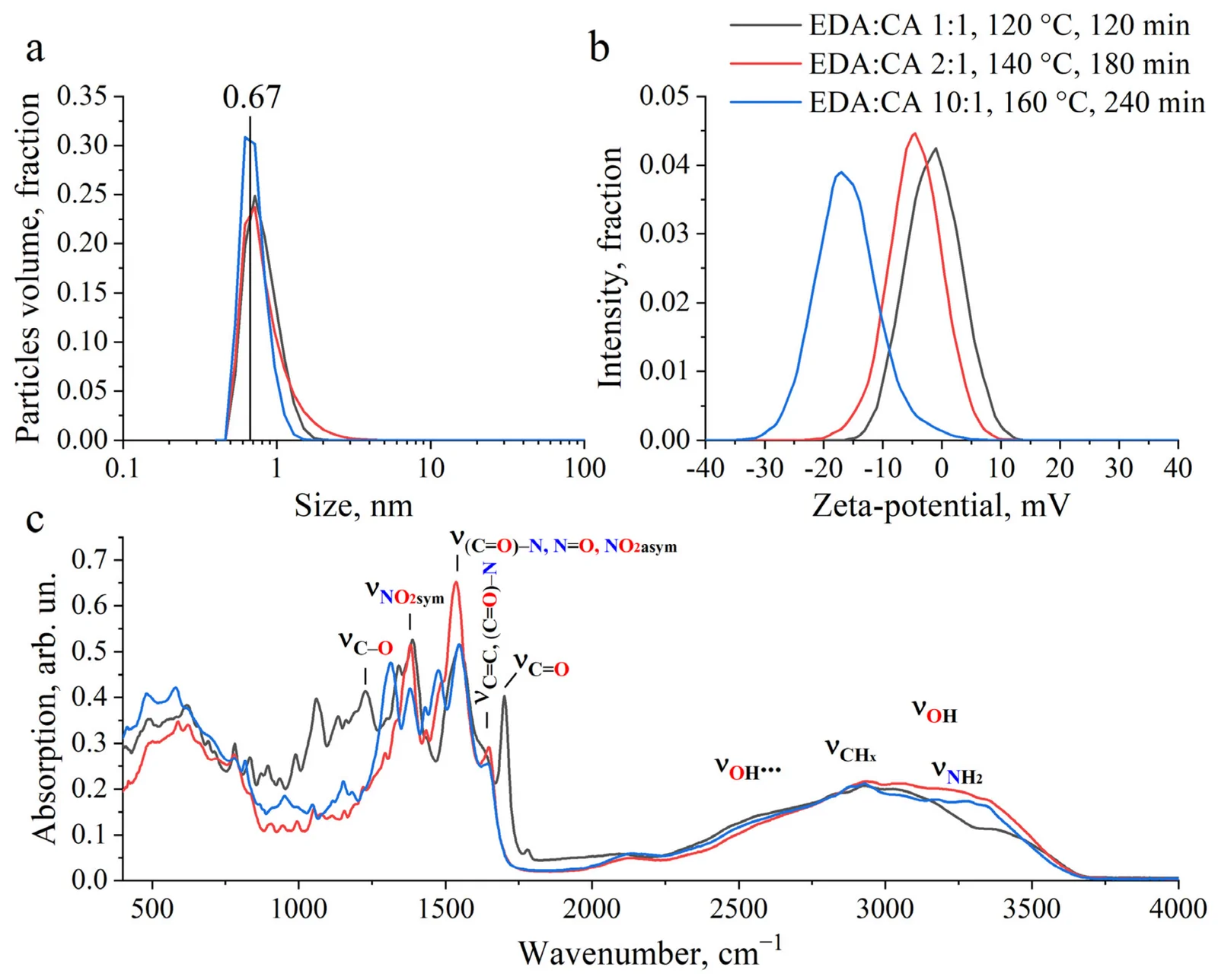
Volume distribution (**a**) and zeta potential (**b**) of aqueous solutions of CDs; FTIR absorption spectra of evaporated CD samples (**c**).

**Figure 2:**
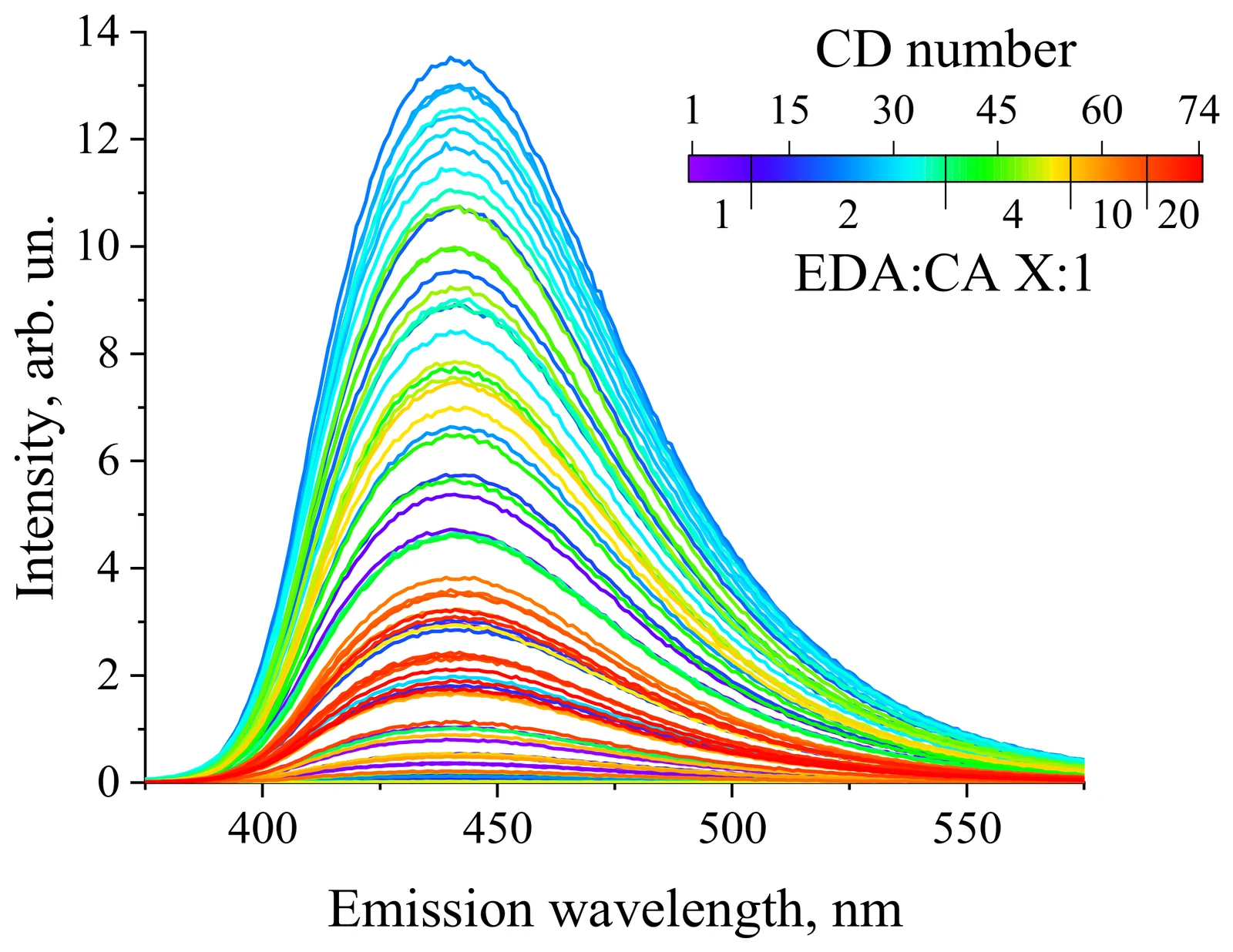
PL spectra of aqueous solutions of CDs synthesized under various conditions in the absence of salts. Signal excitation at a wavelength of 350 nm.

**Figure 3:**
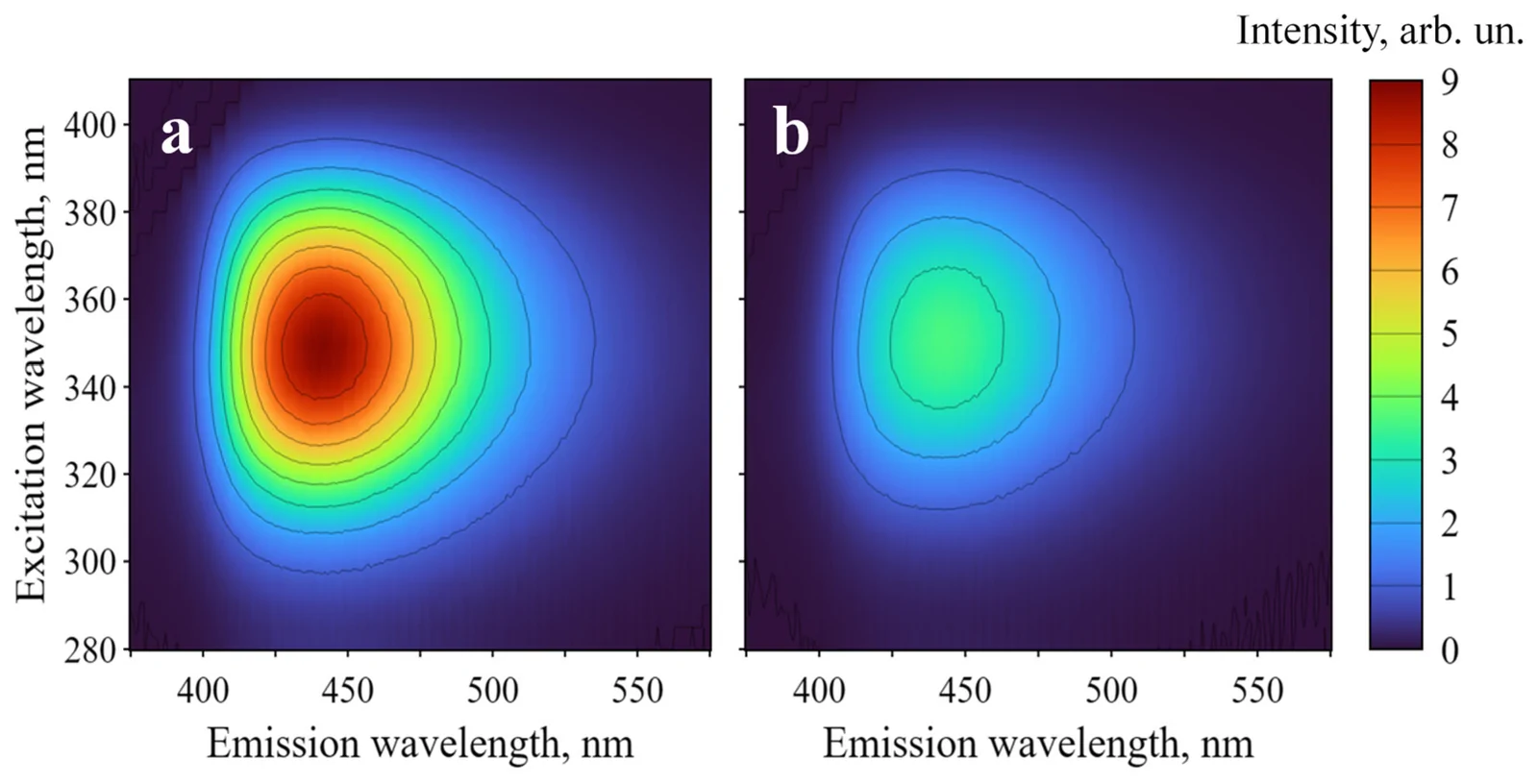
PL excitation–emission matrices of aqueous solutions of CDs (5 mg/L) in the absence of salts (**a**) and in the presence of Co(NO_3_)_2_ and Cu(NO_3_)_2_ salts (each with a concentration of 4 mM) (**b**). The matrices correspond to CDs synthesized with a CA:EDA ratio of 1:2 at 200 °C for 180 min.

**Figure 4:**
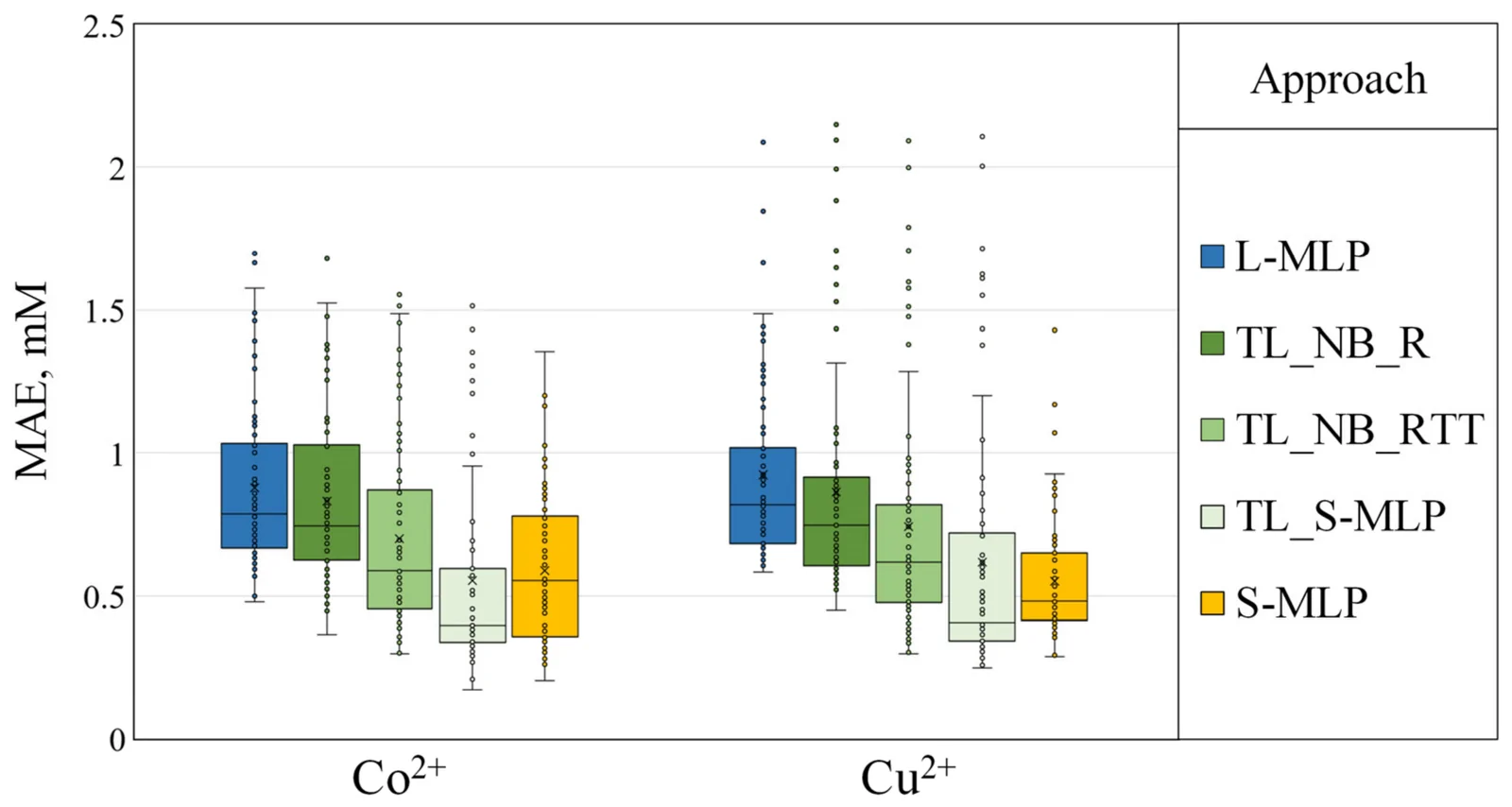
Range diagrams of the average error rates for determining the concentration of Co^2+^ and Cu^2+^ ions using the MLP for all inverse problem formulations. Each diagram reflects the distribution of errors across all types of CDs. The dots represent the average error rates for specific CD types; the line segment shows the range between the 5th and 95th percentiles; and the boundaries and midpoint of the “box” correspond to the 1st and 3rd quartiles and the median value. x represents the arithmetic mean, the horizontal line inside the “box” is the median value.

**Figure 5:**
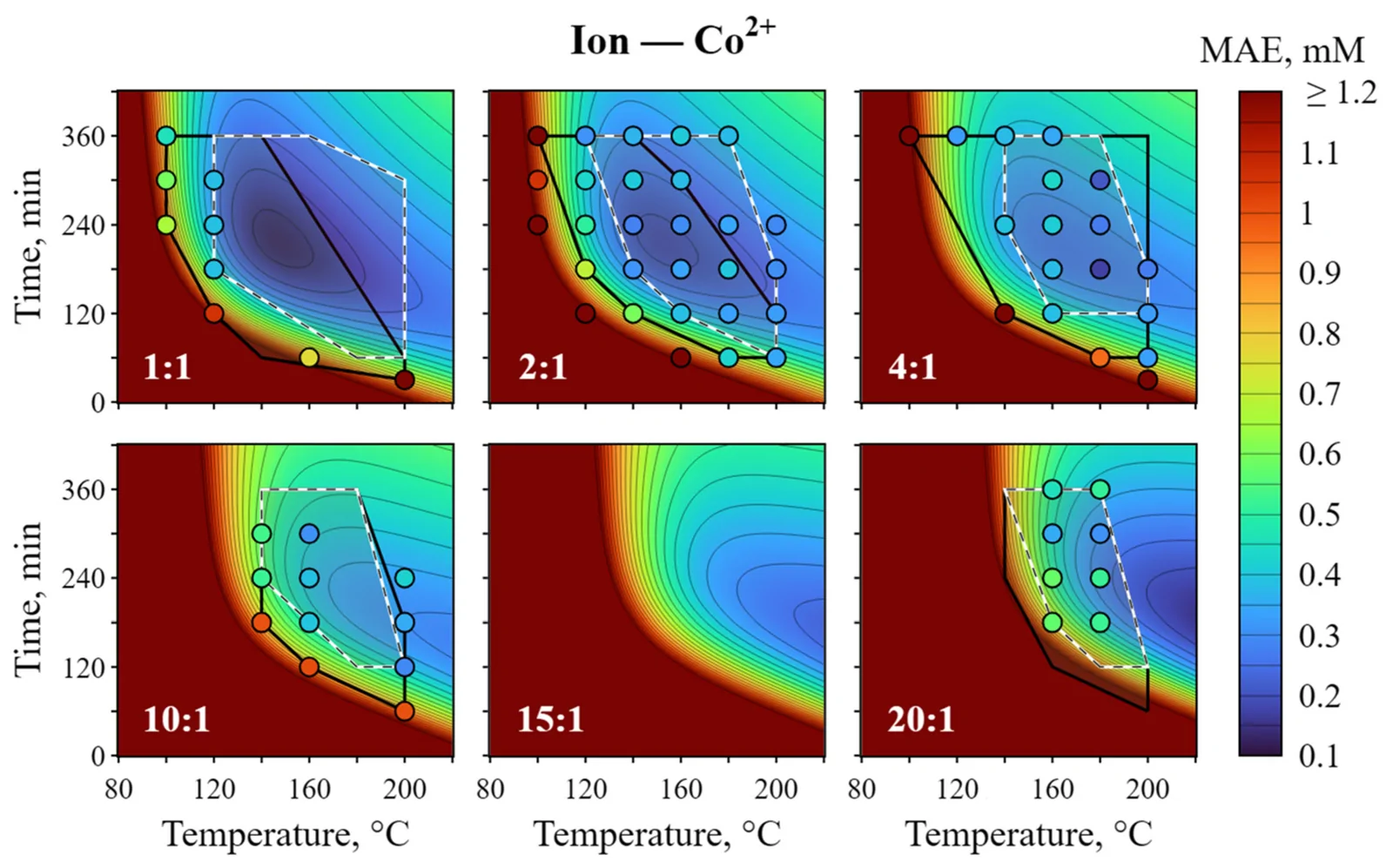
Neural network approximation of the dependence of the MAE for determining the Co^2+^ ion concentration on the CD synthesis conditions: precursor ratio, temperature, and reaction time. The MAE value is shown by the color scale. The circles correspond to the experimental values of the CD synthesis parameters. The color of the circles reflects the MAE value obtained using TL_S-MLP for the corresponding CD type during the error estimation stage. The black solid and white dashed lines delimit the regions of high values of the LQY and of the integrated PL intensity of the CDs, respectively.

**Figure 6:**
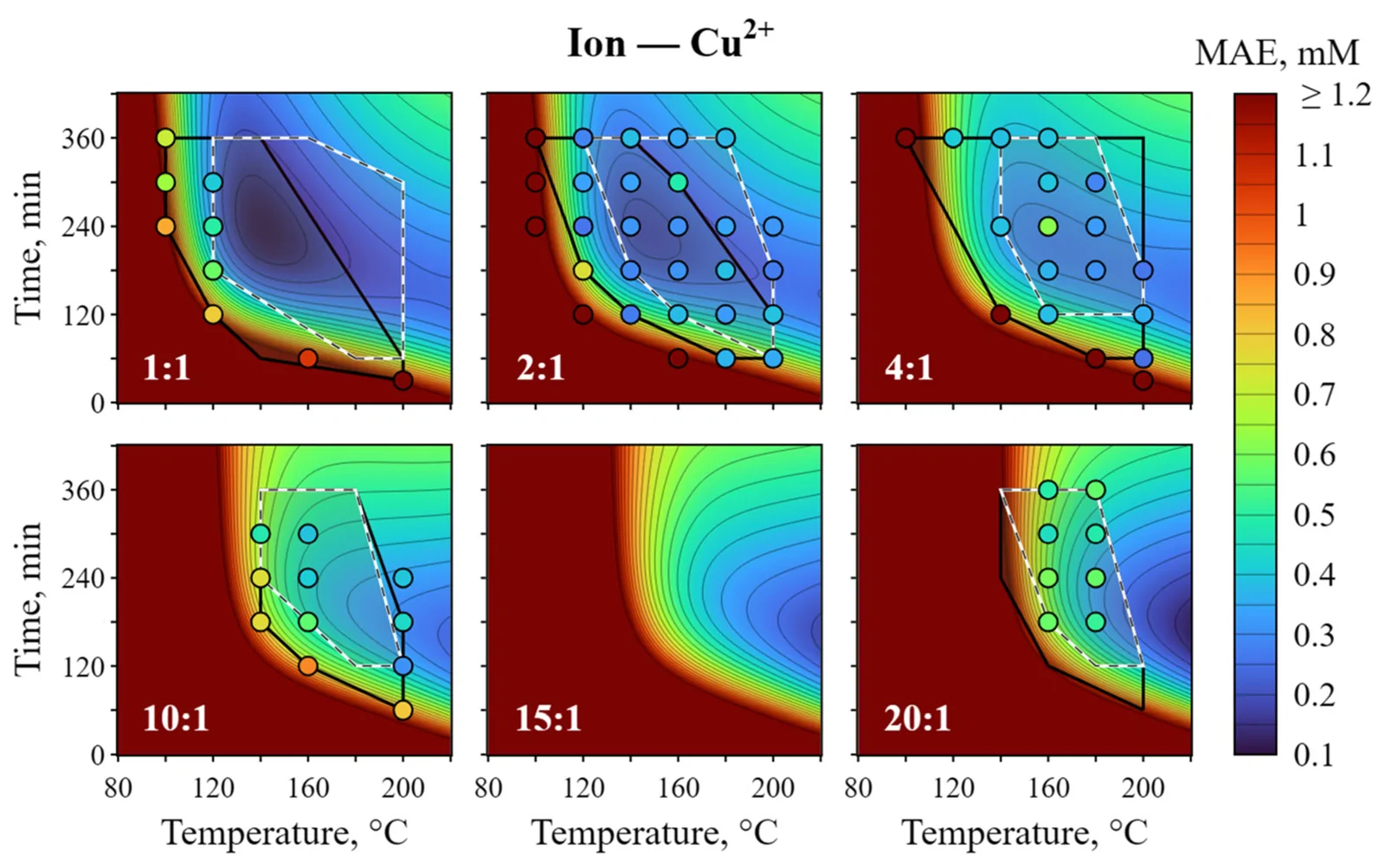
Neural network approximation of the dependence of the error in determining the Cu^2+^ ion concentration on the CD synthesis conditions: precursor ratio, temperature, and reaction time. The MAE value is shown by the color scale. The circles correspond to the experimental values of the CD synthesis parameters. The color of the circles reflects the MAE value obtained using TL_S-MLP for the corresponding CD type during the error estimation stage. The black solid and white dashed lines delimit the regions of high values of the LQY and the integrated PL intensity of the CDs, respectively.

**Figure 7:**
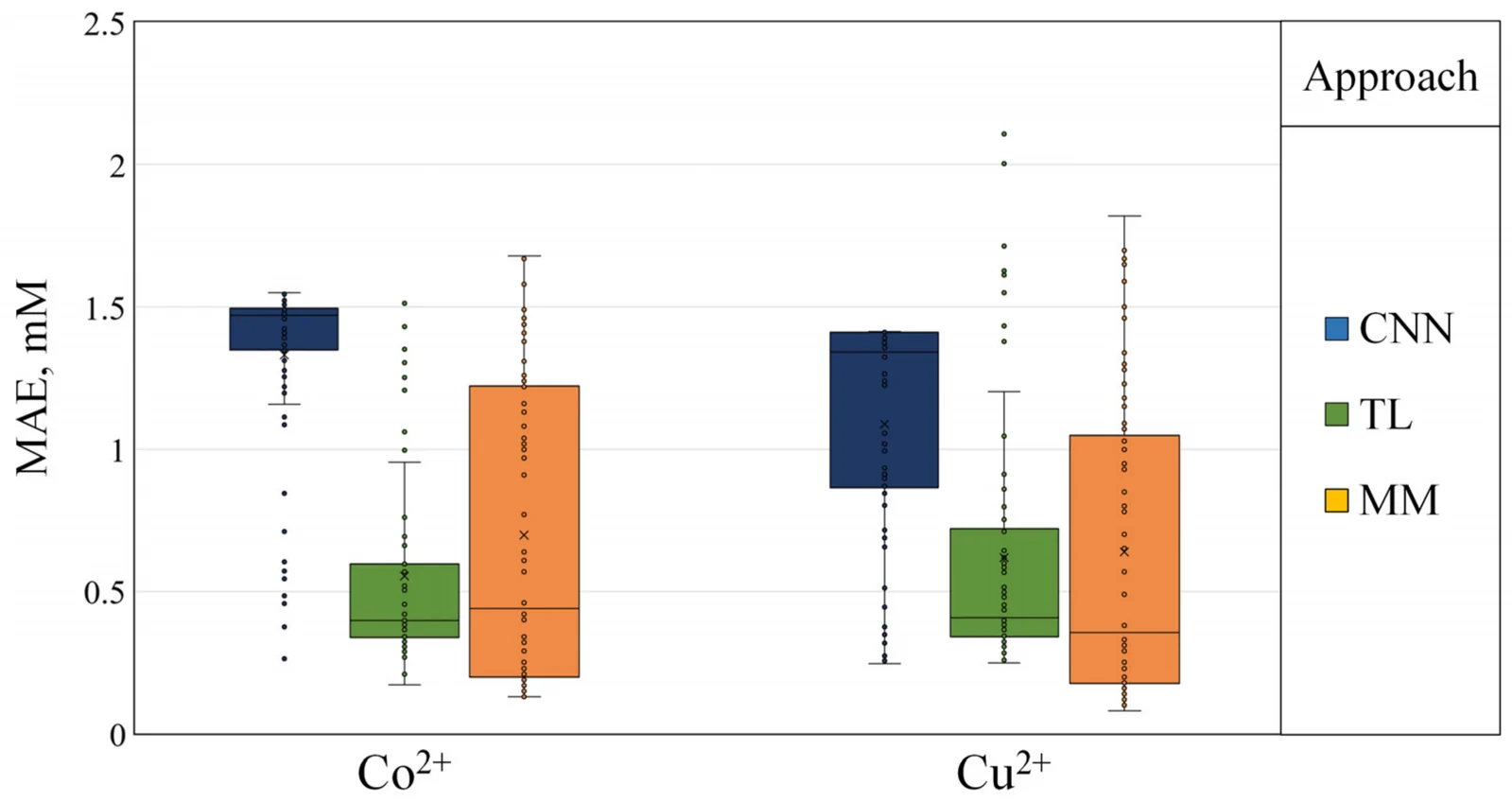
Error range plots for Co^2+^ and Cu^2+^ ion concentration determination obtained using convolutional neural networks (CNN), multi-stage transfer learning (TL), and LR/GB moment-based learning (MM) methods. Each plot reflects the error distribution across all CD types (a description of the display method is provided in the caption to Figure 4).

**Figure 8:**
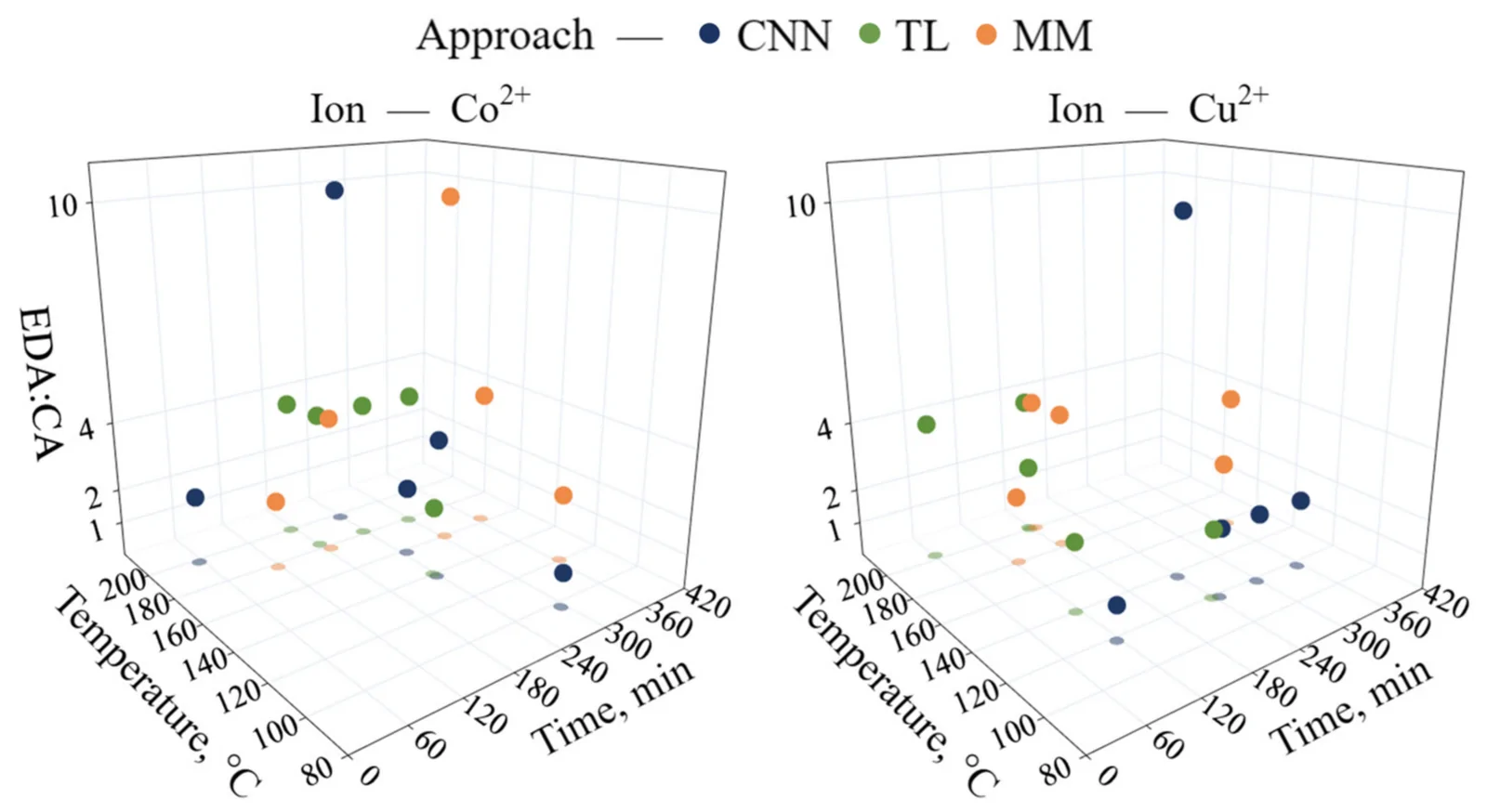
Arrangement of the conditions for the synthesis of CDs in three-dimensional space, ensuring minimal MAE in determining the concentrations of Co^2+^ and Cu^2+^ ions.

**Figure 9:**
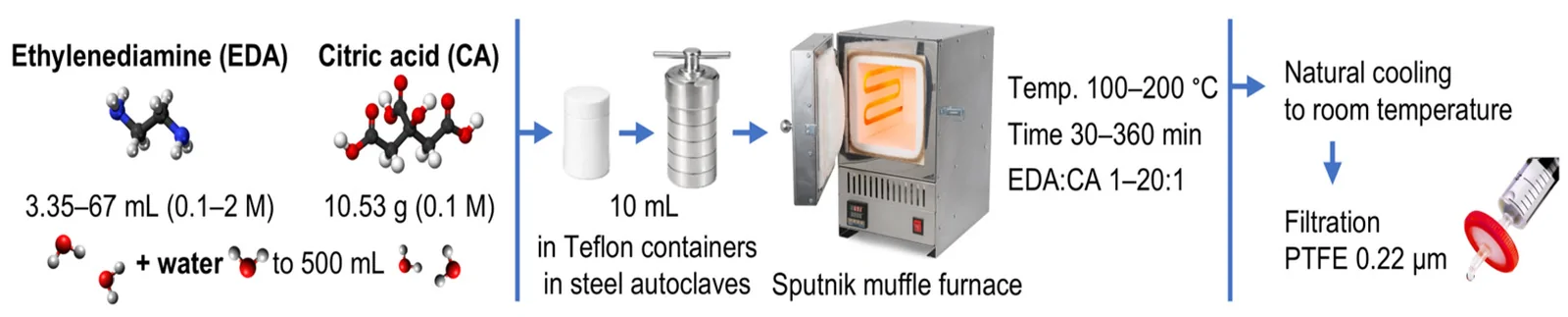
Flowchart of the synthesis process.

**Figure 10:**
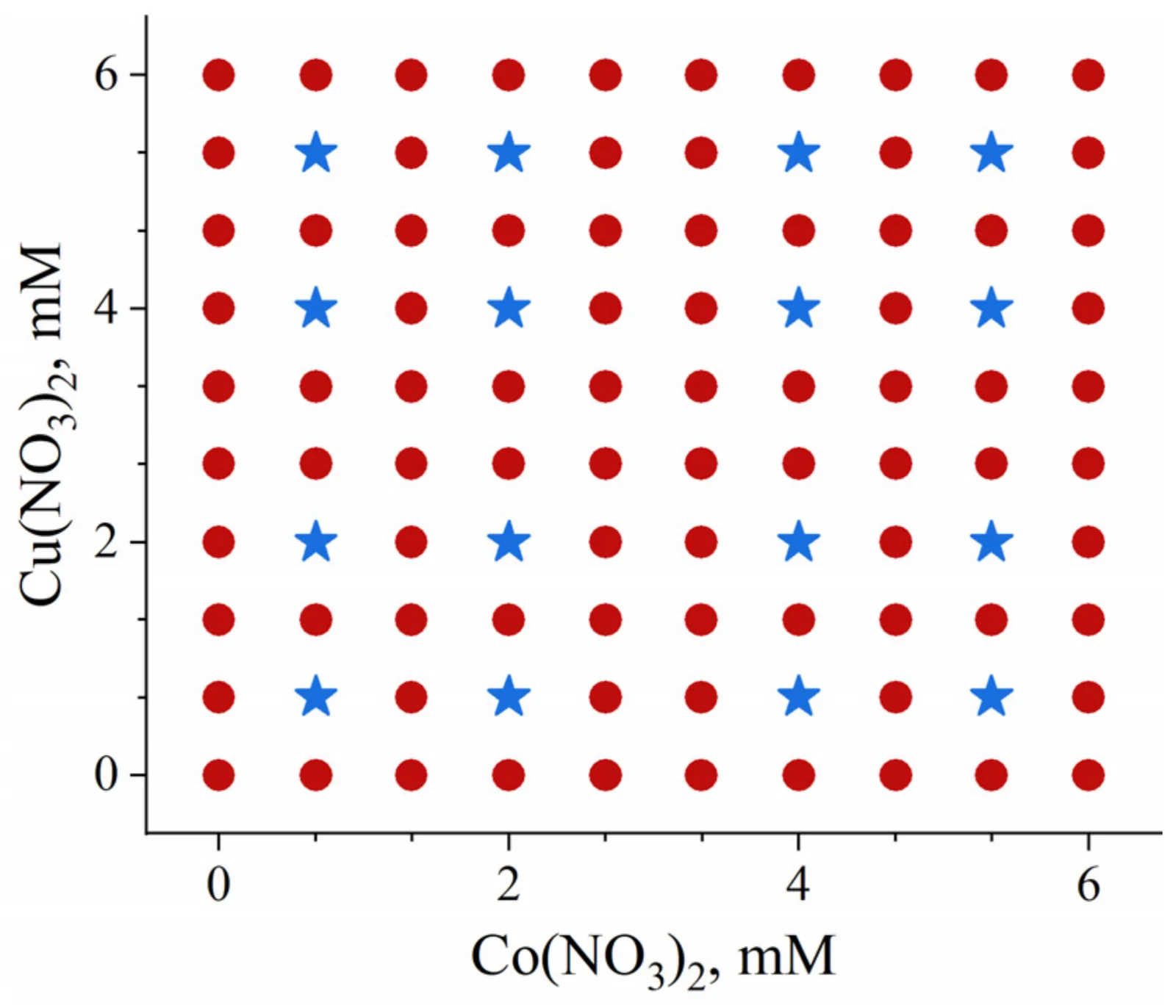
Schematic diagram of the deterministic partitioning of the initial dataset for a specific CD type into training (circles) and test (stars) sets. The points correspond to the experimental grid of concentrations (10 × 10) of the studied heavy metal salts in solutions.

**Figure 11:**
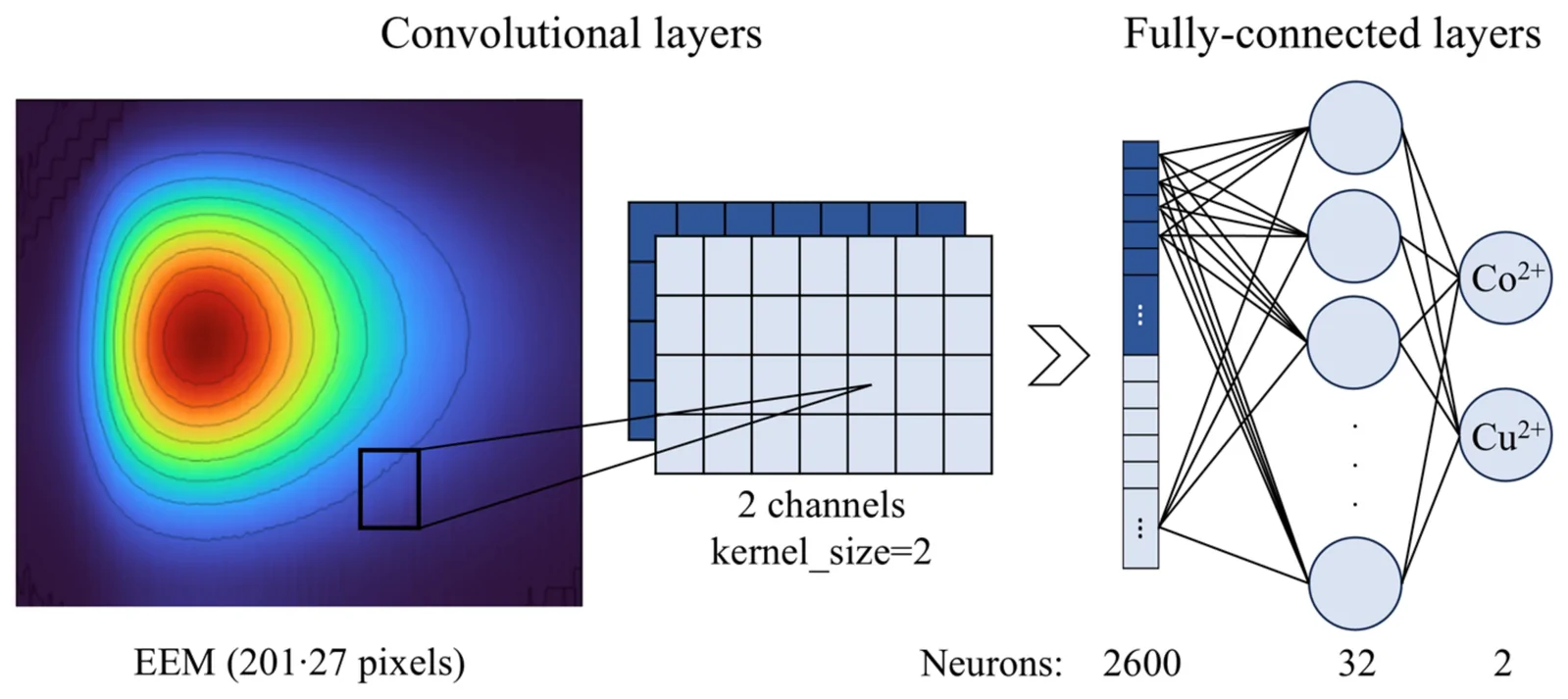
Architecture of the PL EEM—CNN with one convolutional layer.

**Table 1:** Minimum MAE for determining the concentrations of the ions under study, obtained using various machine learning methods. The lowest values for each ion are shown in bold.

Ion	Approach	Synthesis Conditions			MAE, mM
		CA:EDA	Temperature, °C	Time, min	
Co^2+^	CNN	1:2	180	240	0.26
	TL_S-MLP	1:4	180	180	0.17
	MM	1:4	160	360	**0.13**
Cu^2+^	CNN	1:2	160	240	0.25
	TL_S-MLP	1:4	200	60	0.25
	MM	1:4	180	180	**0.08**

**Table 2:** Minimum, median and mean errors in determining the concentrations of the ions under study (in mM), calculated for all types of CDs, for different problem statements. The lowest values for each ion are shown in bold.

Ion	Problem Statement	Minimum Error	Median Error	Mean Error
Co^2+^	L-MLP	0.48	0.79	0.88
	TL_NB_R	0.37	0.74	0.83
	TL_NB_RTT	0.30	0.59	0.70
	TL_S-MLP	**0.17**	**0.40**	**0.55**
	S-MLP	0.20	0.55	0.59
Cu^2+^	L-MLP	0.58	0.82	0.92
	TL_NB_R	0.45	0.75	0.87
	TL_NB_RTT	0.30	0.62	0.74
	TL_S-MLP	**0.25**	**0.41**	0.62
	S-MLP	0.29	0.48	**0.55**

**Table 3:** Mean, median, and minimum MAE for determining the concentrations of the ions under study, calculated from the PL spectra of all types of CDs using CNN, TL_S-MLP, and LR/GB (MM). The lowest values for each ion are shown in bold.

Ion	Method	Minimum Error	Median Error	Mean Error
Co^2+^	CNN	0.26	1.47	1.33
	TL_S-MLP	0.17	**0.40**	**0.55**
	LR/GB (MM)	**0.13**	0.44	0.70
Cu^2+^	CNN	0.25	1.34	1.09
	TL_S-MLP	0.25	0.41	**0.62**
	LR/GB (MM)	**0.08**	**0.36**	0.64

**Table 4:** Optimal conditions for the synthesis of CDs for determining the concentration of the studied ions with the lowest MAE. Results of approximation models.

Ion	Synthesis Conditions			MAE
	CA:EDA	Temperature, °C	Time, min	
Co^2+^	1:2	154	215	0.19
Cu^2+^	1:2	152	220	0.19

**Table 5:** The results of the approbation (MAE for solving the inverse problem of determining the concentrations of ions, mM) near the minima of the approximation models (AMs). The superscripts in the CD sample numbers denote the batch numbers.

CD Sample Number	Co^2+^		Cu^2+^	
	AM Estimation	Experiment	AM Estimation	Experiment
^1^CD1	0.188	0.213	0.188	0.219
^1^CD2	0.190	0.189	0.189	0.192
^2^CD1	0.186	0.199	0.191	0.223
^2^CD2	0.195	0.192	0.191	0.196
^3^CD1	0.191	0.205	0.186	0.211
^3^CD2	0.188	0.194	0.184	0.190

## Data Availability

The raw data supporting the conclusions of this article will be made available by the authors on request.

## Supplementary Materials

The following supporting information can be downloaded at: https://www.mdpi.com/article/10.3390/molecules31213875/s1, Table S1: Values of hyperparameters of models that vary between problem formulations; Table S2: MAE for determining ion concentration using 74 CNNs, each of which was trained on a dataset corresponding to one type of CD; Table S3: MAE value for determining the ion concentration obtained using the MLP on the full dataset (L-MLP) for each type of CD; Table S4: The five lowest MAE values for determining the concentrations of Co^2+^ and Cu^2+^ ions, obtained using CNN, TL-S-MSP, and LR/GB (MM).

## Abbreviations

The following abbreviations are used in this manuscript:

AM	approximation model
CDs	carbon dots
DT	decision trees
EDA	ethylenediamine
EEMs	excitation–emission matrices
XGBoost	extreme gradient boosting
IP	inverse problems
GB	gradient boosting
KNN	K-nearest neighbors
LR	linear regression
LQY	luminescence quantum yield
MLM	machine learning methods
MPCs	maximum permissible concentrations
MAE	mean absolute error
MM	method of moments
MLP	multilayer perceptron
NIR	near-infrared
1D-CNN	one-dimensional convolutional neural networks
OS	optical spectroscopy
PLS	partial least squares
PBQ	p-benzoquinone
PL	photoluminescence
RF	random forest
SVM	support vector machines
TL	transfer learning
