# Supplementary material for: Intelligent Control of the Synthesis of Carbon Dots Used to Create a High-Precision Luminescent Nanosensor of Heavy Metal Ions in Water Using Machine Learning
Source: Molecules. 2026 Oct 27;31(21):3875. doi: 10.3390/molecules31213875 (PMC13765085; doi:10.3390/molecules31213875)
Supplement: Supplementary file 1 [file molecules-31-03875-s001.zip › molecules-4644919-supplementary.pdf]

molecules

Intelligent control of carbon dot synthesis to create a high-precision luminescent nanosensor for heavy metal ions in water using machine learning.

Kirill Laptinskiy, Artem Guskov, Gavriil Kupriyanov, Galina Chugreeva, Alexey Vervald, Sergey Dolenko, Tatiana Dolenko

Table S1. Values of hyperparameters of models that vary between problem formulations.

Problem statement	The proportion of training data allocated for the validation set	Learning rate	Ratio L2 regularization
L-MLP	0.1	0.001	0.0001
TL_NB_R	0.1	0.002	0.00025
TL_NB_RTT	0.1	0.0005	0.0005
TL_S-MLP	0.19 (16 examples)	0.0001	0.00075
S-MLP	0.19 (16 examples)	0.0005	0.00075

Table S2. MAE for determining ion concentration using 74 CNN, each of which was trained on a dataset corresponding to one type of CD.

Sample	Synthesis conditions			MAE, mM	
	CA:EDA	Temperature, °C	Reaction time, min	Co^{2+}	Cu^{2+}
1	1	100	240	0.94	0.83
2	1	100	300	0.42	0.28
3	1	100	360	1.03	0.74
4	1	120	120	1.53	0.80
5	1	120	180	1.04	0.74
6	1	120	240	1.00	0.80
7	1	120	300	0.97	0.87
8	1	160	60	0.95	0.85
9	1	200	30	1.40	1.54
10	2	100	240	0.98	0.83
11	2	100	300	0.99	0.92
12	2	100	360	0.84	1.34
13	2	120	120	1.37	1.34
14	2	120	180	0.90	1.32
15	2	120	240	0.79	1.30

16	2	120	300	0.43	1.36
17	2	120	360	1.29	1.33
18	2	140	120	0.44	1.45
19	2	140	180	0.95	0.76
20	2	140	240	0.79	0.50
21	2	140	300	1.34	1.33
22	2	140	360	1.36	0.76
23	2	160	60	1.30	1.61
24	2	160	120	1.31	1.37
25	2	160	180	0.95	0.84
26	2	160	240	1.27	0.25
27	2	160	300	0.99	0.79
28	2	160	360	0.72	1.34
29	2	180	60	1.22	0.91
30	2	180	120	1.33	0.75
31	2	180	180	1.56	1.32
32	2	180	240	0.26	1.30
33	2	180	360	0.72	1.33
34	2	200	60	0.94	0.80
35	2	200	120	1.33	0.95
36	2	200	180	1.26	0.78
37	2	200	240	1.30	0.84
38	4	100	360	0.76	1.31
39	4	120	360	1.23	0.87
40	4	140	120	1.31	0.67
41	4	140	240	0.76	1.29
42	4	140	360	0.98	0.85
43	4	160	120	1.45	1.43
44	4	160	180	1.29	1.18
45	4	160	240	1.02	0.93
46	4	160	300	1.37	0.87
47	4	160	360	1.36	0.69
48	4	180	60	0.86	0.51
49	4	180	180	0.86	1.19
50	4	180	240	1.18	0.85
51	4	180	300	0.94	0.89
52	4	200	30	0.87	0.99
53	4	200	60	0.97	0.91
54	4	200	120	1.33	1.31
55	4	200	180	0.69	1.29
56	10	140	180	1.40	0.91
57	10	140	240	0.98	0.93
58	10	140	300	1.36	0.75
59	10	160	120	0.61	0.92

60	10	160	180	1.16	0.66
61	10	160	240	0.97	1.27
62	10	160	300	1.17	1.29
63	10	200	60	1.04	0.80
64	10	200	120	0.93	0.88
65	10	200	180	0.56	1.37
66	10	200	240	1.35	1.20
67	20	160	180	0.91	0.79
68	20	160	240	1.25	0.98
69	20	160	300	0.80	0.96
70	20	160	360	1.34	1.35
71	20	180	180	1.31	1.31
72	20	180	240	1.25	1.31
73	20	180	300	0.78	1.00
74	20	180	360	1.33	1.58

Table S3. MAE determinations of ion concentration obtained using the MLP on the full data set (L-MLP) for each type of CD.

	Synthesis conditions			MAE, mM	
**Sample**	**CA:EDA**	**Temperature, °C**	**Reaction time, min**	**Co^{2+}**	**Cu^{2+}**
1	1	100	240	0.96	1.09
2	1	100	300	0.75	0.89
3	1	100	360	0.85	0.96
4	1	120	120	1.11	0.92
5	1	120	180	0.75	0.95
6	1	120	240	1.02	0.89
7	1	120	300	0.72	0.81
8	1	160	60	1.12	1.16
9	1	200	30	1.35	2.09
10	2	100	240	1.7	1.42
11	2	100	300	1.11	1.85
12	2	100	360	1.46	1.31
13	2	120	120	1.49	1.49
14	2	120	180	0.89	1.29
15	2	120	240	0.79	1.01
16	2	120	300	0.82	0.83
17	2	120	360	0.5	0.81
18	2	140	120	0.84	0.96
19	2	140	180	0.95	0.73

20	2	140	240	0.64	0.68
21	2	140	300	0.7	0.64
22	2	140	360	0.63	0.80
23	2	160	60	1.34	1.45
24	2	160	120	1.39	0.83
25	2	160	180	0.75	0.63
26	2	160	240	0.59	0.63
27	2	160	300	0.69	0.85
28	2	160	360	0.72	0.76
29	2	180	60	0.72	1.07
30	2	180	120	0.71	0.68
31	2	180	180	0.64	0.63
32	2	180	240	0.75	0.68
33	2	180	360	0.61	0.61
34	2	200	60	1.3	0.82
35	2	200	120	0.86	0.9
36	2	200	180	0.65	0.66
37	2	200	240	0.71	0.8
38	4	100	360	1.58	1.85
39	4	120	360	0.78	0.58
40	4	140	120	1.39	1.44
41	4	140	240	0.83	0.63
42	4	140	360	1.02	0.91
43	4	160	120	0.92	0.61
44	4	160	180	0.63	0.69
45	4	160	240	0.64	1.27
46	4	160	300	0.82	1.19
47	4	160	360	0.65	1.24
48	4	180	60	1.18	1.39
49	4	180	180	0.78	0.65
50	4	180	240	0.69	0.81
51	4	180	300	0.58	0.78
52	4	200	30	1.66	1.67
53	4	200	60	1.06	0.62
54	4	200	120	0.7	0.61
55	4	200	180	0.61	0.65
56	10	140	180	1.13	0.9
57	10	140	240	0.59	0.85
58	10	140	300	0.96	0.74
59	10	160	120	1.09	0.93
60	10	160	180	0.72	0.72
61	10	160	240	1	0.67
62	10	160	300	0.79	0.77
63	10	200	60	1.07	0.85

64	10	200	120	0.61	0.85
65	10	200	180	0.64	0.65
66	10	200	240	0.83	0.63
67	20	160	180	0.8	0.99
68	20	160	240	0.91	0.79
69	20	160	300	0.58	0.74
70	20	160	360	0.57	0.76
71	20	180	180	0.81	1.02
72	20	180	240	0.67	0.73
73	20	180	300	0.48	0.79
74	20	180	360	0.73	0.80

Table S4. The five lowest MAE values for determining the concentrations of Co^{2+} and Cu^{2+} ions, obtained using CNN, TL-S-MSP, and LR/GB (MM).

Ions	Approach	Synthesis conditions			Errors in the solution of the inverse problem
		CA:EDA	Temperature, °C	Reaction time, min	
Co^{2+}	CNN	1:2	160	240	0.26
		1:2	200	60	0.38
		1:1	100	300	0.46
		1:10	200	240	0.49
		1:4	140	240	0.54
	TL	1:4	180	180	0.17
		1:4	180	300	0.21
		1:4	180	240	0.27
		1:4	200	180	0.27
		1:2	140	240	0.28
	MM	**1:4**	**160**	**360**	**0.13**
		1:2	180	120	0.14
		1:10	160	300	0.14
		1:4	180	180	0.15
		1:2	120	360	0.16
Cu^{2+}	CNN	1:2	120	240	0.25
		1:1	120	120	0.26
		1:2	120	360	0.27
		1:10	140	240	0.32
		1:2	120	300	0.33
	TL	1:4	200	60	0.25
		1:2	120	240	0.26
		1:4	200	180	0.26

	1:2	140	120	0.27
	1:2	200	180	0.27
	**1:4**	**180**	**180**	**0.08**
	1:2	160	360	0.10
MM	1:4	200	180	0.12
	1:4	160	360	0.12
	1:2	180	120	0.13
